# Phenotypic and Molecular Diversity of *Salicornia bigelovii* and Its Potential for Salinity-Resilient Industrial Oilseed Production

**DOI:** 10.3390/plants15203210

**Published:** 2026-10-19

**Authors:** Jawaher AlYammahi, Houda Chelaifa, Bushra G. Mohammed, Hector H. Hernandez

**Affiliations:** 1 Department of Chemical and Petroleum Engineering, Khalifa University, Abu Dhabi P.O. Box 127788, United Arab Emirates; 2 Sustainable Bioenergy Research Consortium, Khalifa University, Abu Dhabi P.O. Box 127788, United Arab Emirates; 3 Division of Engineering, New York University Abu Dhabi, Abu Dhabi P.O. Box 129188, United Arab Emirates; 4 Crop Diversification and Genetics Program, International Center for Biosaline Agriculture (ICBA), Dubai P.O. Box 14660, United Arab Emirates; 5 Department of Pharmaceutical Chemistry, Shadan Collage of Pharmacy, Hyderabad 500091, India

**Keywords:** *Salicornia bigelovii*, seeds, halophyte, phylogeny, phenotypes, morphology

## Abstract

*Salicornia bigelovii* is a promising oilseed crop known for its high yield potential and exceptional salt tolerance, ranking among the most salt-resistant vascular plants. Although previous studies have investigated individual phenotypic and genetic characteristics of *S. bigelovii*, integrated assessments combining agronomic performance, seed morphometric variation, and genetic relationships among geographically distinct accessions remain limited. This study evaluated six *S. bigelovii* accessions from the Texas Gulf coastline, focusing on phenotypic and morphometric traits, geographic origins, and genomic distances. Significant variability was observed among accessions in key traits, such as plant height, spike number, root length, and seed yield. Notably, the South Padre and Boca Chica accessions demonstrated superior growth and higher seed productivity compared with Corpus Christi Bridge and Galveston accessions, indicating substantial phenotypic diversity and potential for selecting high-performing accessions for breeding. Morphological analysis revealed minor but distinct differences among the six seed accessions. The southern accessions, SPN and SP, exhibited advantageous traits, including a high surface area, circularity values close to 1, and increased seed volume, suggesting favorable dispersal and flow characteristics. Multivariate analyses and hierarchal clustering showed significant relationships among accessions, providing insights for breeding programs. Phylogenetic analysis indicated the divergence of CCB and G accessions from BC, BCW, SP, and SPN. This morphological and genetic assessment establishes a foundation for accelerated molecular breeding and genomic-assisted improvement of *S. bigelovii* as an industrial crop.

## 1. Introduction

The halophyte *Salicornia bigelovii*, commonly known as dwarf saltwort, pickle weed, or dwarf glasswort, is one of the world’s most salt-tolerant land vascular plants [1,2]. *Salicornia bigelovii* is a tetraploid species of flowering plant in the family Chenopodiaceae and subfamily Salicornioideae [3]. This crop exhibits potential as a seawater agronomic resource, offering multiple applications, including fiber production from biomass, silage for feed, fuel derived from seed oil, protein-rich meal post oil extraction, and human consumption of fresh stems [4,5]. Previous studies, including our own, have demonstrated the potential of *S. bigelovii* seed oil as a feedstock for biofuel production [6,7,8].

*S*. *bigelovii* plants are found in the United States with broad geographical distribution, thriving in harsh coastal environments such as salt flats, margins of wind tidal flats, and the margins of hurricane wash over channels [9]. It has been evaluated as an oilseed crop along the desert coastline of Mexico and is found in greater abundance along the Gulf of Mexico, stretching from the Yucatan Peninsula north to Texas and east to Florida and the Florida Keys [10]. In recent years, *S. bigelovii* has been cultivated in many countries around the world for various purposes. For example, *S. bigelovii* was grown in the UAE as part of a pilot initiative known as the Seawater Energy and Agriculture System (SEAS) aimed at producing aviation biofuel [7]. Nonetheless, the large-scale cultivation and breeding of *S. bigelovii* are challenged by the limited characterization of key morphological traits relevant to crop production. These include variation in seed size and shape, which may influence seed metering and sowing uniformity during mechanized planting, differences in root system architecture that may affect plant establishment and performance, and insufficient information on phenotypic plasticity among accessions across different growing environments.

Seed morphology has proven instrumental in analyzing taxonomic relationships across a diverse range of plant families and genera. It facilitates the quantification of morphological variation, encompassing changes during imbibition, differences among related accessions, and alterations in shape in response to environmental factors [11,12]. Seed shape and seed size are considered to be valuable agronomic traits reflecting genetic, physiological, and ecological components, which also affect the yield and quality of the bio-oil derived from these seeds [13]. Digital image analysis, along with microscopic techniques for the seed’s shape and size quantification, provides a powerful tool to understand the structure–property–functionality relationship of oilseeds [14]. Moreover, seed size and shape features are crucial in terms of designing the seed metering mechanism of seed drills, transportation, separation and sizing systems [15,16]. Documenting these traits across accessions is therefore relevant to breeding programs, as seed size and shape can serve as potential selection criteria for improving seed uniformity and other desirable characteristics, while facilitating the optimization of mechanized handling, metering, sowing, and processing. In addition, seed morphology is closely associated with seed development and may influence agronomic performance and seed quality characteristics. The characterization of seed phenotypic variation among available germplasm is an important step toward understanding the diversity of this emerging halophytic crop.

In addition to morphological characterization, molecular characterization is essential for assessing the level of genetic diversity present among the different accessions of the same *Salicornia* species. Several molecular phylogenetic studies using nuclear and chloroplast DNA sequences have been conducted on the *Salicornia* genus, in an attempt to establish the genetic diversity and phylogenetic relationships in the *Chenopodiaceae* family [9,17,18,19,20]. Currently, the *Salicornia* genus comprises 25 to 30 species, although there are questions regarding the total accepted species because numerous species and aggregates have been described over the last 250 years [21]. The challenges associated with resolving the taxonomy of the *Salicornia* genus stem from several factors, namely, scarcity of distinct plant morphological characteristics, an insufficient supply of preserved material for taxonomic studies, phenotypic plasticity influenced by their habitats, and the provision of conflicting information by regional studies due to the lack of a comprehensive global genetic resource [21]. Kadereit et al. presented the *Salicornia* lineage based on ETS sequences, in which the *S*. *bigelovii* clade formed a polytomy with decaploid species and seven other accessions from Central Asia of unknown ploidy level, indicating the need for further molecular phylogenetic studies on *S*. *bigelovii* species to resolve its phylogeny [22]. Whether the genetic divergence detected at higher taxonomic levels is also reflected among individual *S. bigelovii* accessions used or considered for cultivation and breeding remains an open question, which we address here using ITS sequence analysis.

The present study focuses on the phenotypic and ITS-based molecular characterization of *S. bigelovii* accessions rather than direct measurements of oil content or fatty acid composition. Therefore, the objective of this study was to characterize six *S*. *bigelovii* accessions cultivated under common environmental conditions. Specifically, we aimed to evaluate variation in agronomic and seed morphometric traits among the accessions, assess seed surface macro-structure, investigate molecular relationships based on ITS sequences, and compare phenotypic and molecular patterns to provide a baseline characterization of the available *S. bigelovii* germplasm for future genetic and breeding studies. We hypothesized that *S. bigelovii* accessions originating from different collection sites along the Texas Gulf coastline would exhibit significant differences in agronomic performance, seed morphology, and ITS sequence variation when grown under identical environmental conditions. Consistent with this hypothesis, the accessions differed significantly in several measured traits, including root architecture, seed size, shape, and physical properties, while ITS-based clustering revealed genetic differentiation among accessions that broadly corresponded with their geographic origins. SP, SPN, and BC also exhibited higher values than CCB and G for several agronomic and seed-related traits. Collectively, these findings provide a baseline phenotypic and genetic characterization of the evaluated *S. bigelovii* accessions to inform future germplasm evaluation and breeding studies.

## 2. Materials and Methods

### 2.1. Sample Collection and Geographical Analysis

*S*. *bigelovii* seeds used in this study were sourced from a germplasm collection maintained at Khalifa University. This collection comprised six distinct accessions of *Salicornia bigelovii* seeds, originally collected from various locations along the Texas Gulf coastline as indicated in Table 1. Although originally collected from six distinct sites along the Texas Gulf Coast, all six *S. bigelovii* accessions were subsequently sown and cultivated together under identical greenhouse conditions at the University of Arizona. Seeds produced from these plants were used for the present study and sown at the Seawater Energy and Agriculture System (SEAS) experimental station in Masdar City, Abu Dhabi, UAE. All accessions were cultivated under the same experimental conditions, irrigated with the same seawater source, and received no compost or soil amendments. Phenotypic characterization was therefore performed on plants raised under a common cultivation environment rather than at their original collection sites.

**Figure 1:**
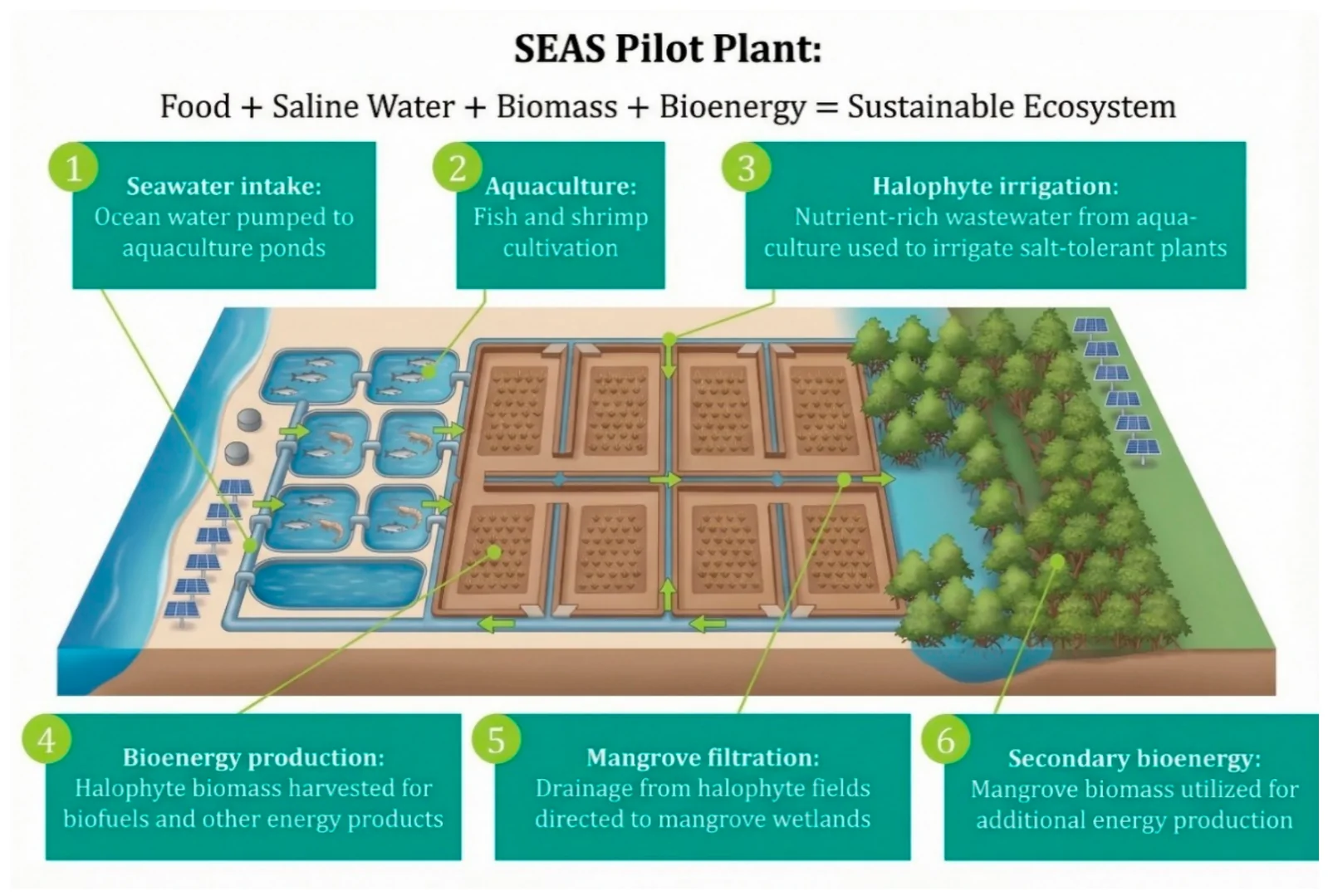
Schematic of the Seawater Energy and Agriculture System (SEAS) pilot facility in Abu Dhabi, UAE, illustrating the three integrated aquaculture and agriculture components.

The SEAS facility consists of three primary components: aquaculture ponds, halophyte fields, and mangrove wetlands, creating a symbiotic ecosystem as shown in Figure 1. The fish and shrimp are cultivated in aquaculture ponds, and the nutrient-laden water from these ponds is utilized to irrigate the halophyte ‘*S. bigelovii*’ fields. Eventually, the water runoff from the halophyte fields is directed to the seawater where mangroves are cultivated. This cutting-edge system not only optimizes the use of land and water but also highlights the potential for sustainable food and bioenergy production in non-arable desert land, utilizing seawater for irrigation. This study acts as an essential foundation for creating an improved breeding program.

To elucidate the geographical distribution of the studied *S*. *bigelovii* accessions, we employed ArcGIS software (Environmental Systems Research Institute, Redlands, CA, USA) to plot the geospatial coordinates of the sampling sites on a map of Texas. This visualization provides crucial insights into the potential environmental influences (such as soil type) on genetic and phenotypic variations observed among the accessions. Table 1 displays the XY coordinates, soil type and soil map.

### 2.2. Plant Phenotypic Characterization

Seeds of six *S*. *bigelovii* accessions were sown at the SEAS experimental station (Masdar City, Abu Dhabi, UAE). The evaluated germplasm consisted of accessions received from the University of Arizona, originally collected from coastal wetlands and salt marsh ecosystems in Texas (Table 1). Prior to sowing, the field was leveled and flooded with effluent water originating from aquaculture ponds, and no compost or soil amendments were applied. Irrigation was carried out using seawater with salinity ranging between approximately 50 and 60 dS·m^−1^. All six accessions were irrigated from the same seawater source and received no compost or soil amendments, ensuring common growing conditions across accessions. Each accession was planted in a separate 645 m^2^ field, divided into six plots of approximately 100 m^2^ each; ten plants were randomly selected from each plot for phenotypic evaluation.

Weekly phenotyping measurements were conducted throughout the growing season to capture temporal variation in morphological and agronomic traits. Quantitative phenotyping included measurements of plant height, number of branches, number of reproductive spikes, and spike length. At harvest, detailed productivity assessments were performed, including measurement of seed weight per plant, fresh and dry above-ground biomass, and root morphological traits such as primary and secondary root length and the number of secondary roots, enabling evaluation of biomass allocation and root–shoot relationships. Table 2 displays the studied plant’s traits along with their abbreviation.

### 2.3. Seed Density Measurement

An automatic Gas Pycnometer (AccuPyc 1340TC, Georgia, USA) with helium as a carrier gas was used to determine the apparent volumes of *S*. *bigelovii* seeds. Volume measurements were repeated ten times for each biological replicate (3 replicates), and mean values were used for calculation of seed density. The mass values of the seeds were determined using an electronic balance reading to 0.001 g.

### 2.4. Digital Stereo Microscope

For each *S*. *bigelovii* accession, approximately 100 mg of seeds were manually cleaned and sieved to remove debris and damaged specimens. A random sample of more than 100 seeds per accession was then imaged using a digital stereo microscope (Thomas Scientific, Swedesboro, NJ, USA) equipped with an RGB camera (Andoer 34MP Full HD, Guangdong, China) at 1× magnification. High-quality images were captured in RGB color and stored in TIFF format (720 × 480 pixels resolution) to preserve detail for subsequent morphometric analysis using ImageJ software. This standardized imaging protocol ensured consistent and accurate representation of seed morphology across all accessions.

### 2.5. Image Analysis

The digital images obtained from the digital light microscope were used to extract detailed morphological features by means of image analysis. Pixel dimensions were set using a calibration slide with known dimensions (Figure S1) and expressed in mm units. RGB images were transformed into an 8-bit grayscale format utilizing the ImageJ 1.43u software [11]. The seed binary images were obtained by manual segmentation from grayscale images using the automatic threshold of the modified IsoData algorithm in ImageJ. Seed shape attributes were determined through the length/width (L/W) ratio analysis [11]. The Bounding Rectangle algorithm, followed by the Fitted Ellipse, was utilized in ImageJ to estimate the length and width dimensions, as well as the parameters listed below [11]. The following morphological features were determined from the binary images:

Feret diameter (FD): The longest distance between two points of the object’s border, also known as maximum caliper.

Area (A): Area of selection in pixels squared and in calibrated units squared (µm^2^).

Perimeter (P): The continuous length of the outside boundary of the seed.

Aspect ratio (AR): The aspect ratio of the particle’s fitted ellipse, which is a relationship between the major and minor axes. A value of 1.0 corresponds to equidimensional objects and tends to infinity for extended objects.

(1) $$AR=\frac{Major\;Axis}{Minor\;Axis}$$

Roundness (R): The ratio of area to the square root of the major axis or the inverse of AR. R determines the roundness of objects. A round object will have a roundness of 1.0, while the more elongated an object, the more R will approach zero.

(2) $$R=\frac{\left[4\times Area\right]}{\pi \times {\left[Major\;axis\right]}^{2}}$$

Circularity: a value of 1.0 indicates a circle. However, as the value approaches 0.0, the object takes on an increasingly elongated shape.

(3) $$Circularity=\frac{4\pi \times Area}{{Perimeter}^{2}}$$

(4) $$Solidity=\frac{Area}{Convex\;area}$$

Volume: The estimated seed volume was determined using the length and width values by the following equation [23]:

(5) $${Volume}_{1}=0.56822{Volume}_{LW}+0.05156{{Volume}_{LW}}^{2}$$

(6) $${Volume}_{LW}=\pi LW^{2}/6$$

where ${Volume}_{LW}$ is the volume calculated based on the length (L) and width (W) values.

### 2.6. Scanning Electron Microscopy (SEM)

Ten mature seeds from each accession were used to study the seed coat using SEM. Seeds were mounted on aluminum stubs with double-sided carbon adhesive tape and coated with gold–palladium to prevent sample charging. The seed surface was analyzed with the Quanta 250 SEM (FEI, Thermo Fisher Scientific, Waltham, MA, USA) at an accelerating voltage of 10 kV. SEM micrographs were captured at three magnifications (100×, 600×, and 1000×) to determine seed coat morphological features. The terminology of Werker [24] and Shepherd [25] was used to describe the seed’s morphological features.

### 2.7. DNA Isolation, Amplification, and Sequencing

DNA was extracted using 6 g of seeds from each *S. bigelovii* accession. The seeds were manually ground in a sterilized mortar and pestle using liquid nitrogen to facilitate the grinding process. Genomic DNA was extracted from seed powder using the DNeasy Plant Mini Kit (Qiagen, Hilden, Germany) according to the manufacturer’s protocol with some modifications to increase the amount of extracted DNA. The nuclear ITS DNA region used for phylogenetic analysis was amplified from total genomic DNA following the established protocol of Shepherd et al. [20], using a Veriti thermal cycler (Applied Biosystems, Thermo Fisher Scientific, Waltham, MA, USA). The nuclear ribosomal ITS was amplified using the forward and reverse primers ITS4 (5′ TCCTCCGCTTATTGATATGC 3′) and ITS5 (5′ GGAAGTAAAAGTCGTAACAAGG 3′) described by [26]. PCR reactions were carried out in a 20 µL final volume. Each tube contained approximately 100 ng of genomic template DNA, 10 mM MgCl_2_, 10 µM of each of the forward and reverse primers, 10 mM of dNTPs, 1X reaction buffer, 2.4% DMSO, and 0.4-unit DNA polymerase. For amplification of the ITS sequence, PCR conditions were as follows: initial denaturation for 5 min at 94 °C, 40 thermal cycles of 1 min at 94 °C, 1 min at 50 °C and 1 min at 72 °C, and a final extension of 10 min at 72 °C. In a few accessions, further optimization was required to obtain the amplified ITS genes. After thermal cycling, 5 µL aliquots of PCR products were analyzed by electrophoresis on 1% agarose gels. The remaining PCR products were purified using the Oligo Clean-up and Concentration kit (Norgen Biotek Corp., Thorold, ON, Canada), according to the manufacturer’s protocol. PCR products were sequenced using sanger sequencing at Eurofins Genomics (Ebersberg, Germany). PCR amplicons were sequenced in both forward and reverse directions utilizing the ITS4 and ITS5 primers. Forward and reverse sequences were trimmed, merged and the consensus sequences were aligned using the BioEdit software suite [27]. The ITS consensus sequences were blasted against sequences deposited in the GenBank database by Basic Local Alignment Search Tool (BLAST) [28]. The total ITS region length ranged from 455 to 700 bases between the six accessions of *S*. *bigelovii*. Given the tetraploid genomic background of *S. bigelovii*, variation among nuclear ribosomal DNA copies may contribute to ITS sequence heterogeneity; however, additional analyses are required to determine whether the observed length differences represent genuine ITS length polymorphism associated with divergent ribosomal DNA copies or differences arising from sequence recovery and processing [29].

### 2.8. Data Analyses of ITS Sequences

Molecular phylogenetic analysis based on ITS sequence similarities was used to assess the phylogenetic relationship between the *S*. *bigelovii* accessions and other Salicornioideae taxa. The ITS sequences of *Halocnemum strobilaceum* and *Allenrolfea occidentalis*, accession numbers AJ577568 and AY181875, were retrieved from NCBI GenBank and used as an outgroup to root the phylogenetic analysis. In addition, *S*. *bigelovii*’s closest models, *Beta vulgaris* and *Amaranthus caudatus*, accession numbers AY858597 and KX090193, respectively, were included in the analysis. The ITS data included 17 previously published sequences selected from BLAST results that are representative of other Salicornieae and Sarcocornia taxa (listed in Table S4). The obtained ITS sequences were aligned using ClustalX [30] multiple sequence alignment in Geneious software v11.0.2 (refer to Supplementary Information). The phylogenetic tree based on ITS data was built using the Neighbor Joining (NJ) and Maximum Likelihood (ML) methods in NGPhylogeny.fr [31]. The confidence of the phylogenetic tree construction was estimated using bootstrap analysis (100 replicates).

### 2.9. Statistical Analysis

Descriptive statistics (mean ± standard deviation) were calculated for all measured traits. Differences among accessions were analyzed using one-way analysis of variance (ANOVA), with accession as a fixed factor, followed by Tukey’s Honestly Significant Difference (HSD) test for pairwise comparisons (*p* < 0.05). Pearson correlation analysis, Principal Component Analysis (PCA), and hierarchical cluster analysis based on standardized trait values were performed using GraphPad Prism 11 and OriginPro 8.0 software to evaluate relationships and classify accessions. Principal components with eigenvalues > 1.0 (Kaiser criterion) were retained for interpretation.

## 3. Results and Discussion

### 3.1. S. bigelovii Phenotypic Characterization

**Figure 2:**
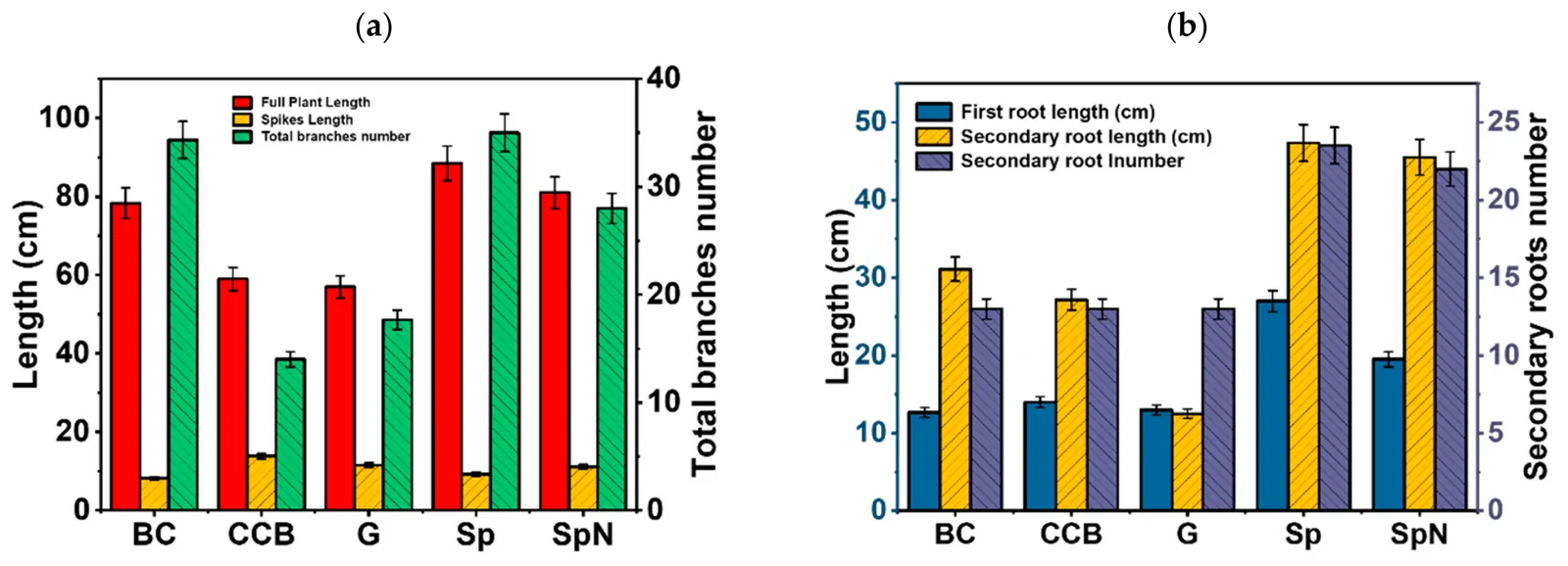

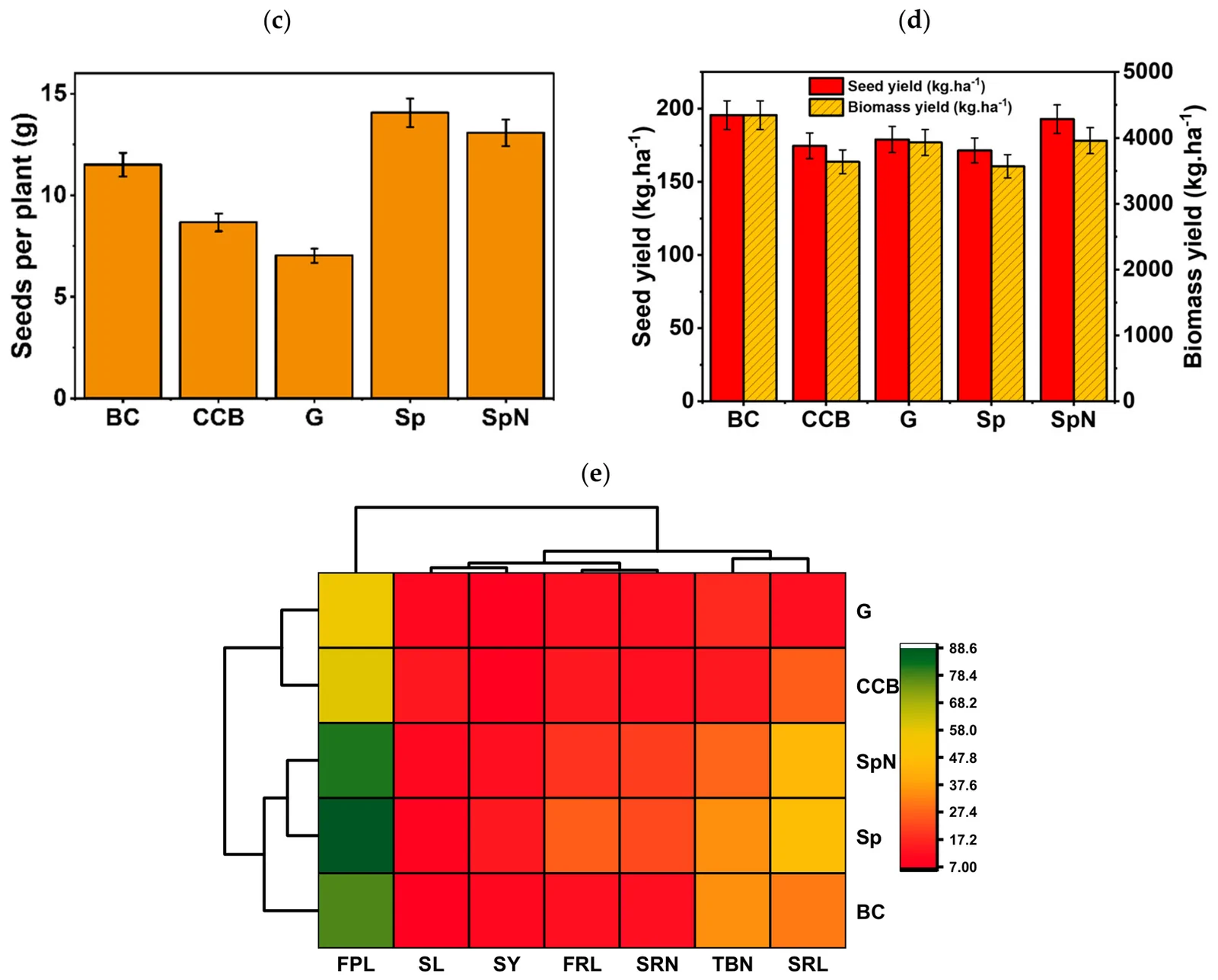
(**a**) Plant height, spike size and spike numbers of five accessions in the SBRC facilities. (**b**) Root length and number of five accessions in the SBRC facilities. (**c**) Seed weight per plant for *S. bigelovii* accessions irrigated with effluent water in the fields. (**d**) Biomass and seed yields of *S*. *bigelovii* accessions. The bar plots display mean values, with error bars indicating the standard deviation (SD). (**e**) Heatmap clustering for all plant phenotypes in the (SEAS) pilot facility.

Throughout the growing season, agronomic traits such as seed germination, plant height, the number of branches and spikes, and spike length were observed over several weeks. At the time of the final harvest, data were gathered from three plants in each field, documenting seed weight per plant, as well as fresh and dry biomass and the morphological characteristics of the roots. All growth parameters of *S. bigelovii* genotypes are presented in Figure 2a,b. The ANOVA test shows statistically significant differences among *S. bigelovii* accessions for spike number (Figure 2a), root length (Figure 2b) and seed weight per plant (Figure 2c). Measurements of plant height exceeding 30 cm were recorded in both BC and SP accessions. Within the facilities, the *S. bigelovii* population was divided into two distinct groups. The first group, consisting of CCB and G, exhibited a lower plant height during their growth phase, measuring between 46 and 55 cm. The heights of these accessions were comparable to those of the *S*. *bigelovii* accessions cultivated at the International Center for Biosaline Agriculture (ICBA), which measured between 48 and 52 cm [11]. The second group includes height measurements greater than 88 cm, which were observed for SP, SPN and BC accessions. These accessions reported higher plant height values as compared with *Sarcocornia* and *Salicornia* species reported by Hayder et al. [32]. The F-test showed statistically significant differences among some accessions. A wider range of variation was observed for number of spikes compared with plant height.

Spike number values showed variations among *S*. *bigelovii* accessions (Figure 2a). BC, SP and SPN accessions received the highest values for spike numbers. The CCB and G plant accessions demonstrated a remarkable difference compared with other accessions. The spike length of *S. bigelovii* plants ranged between 60 mm and 128 mm, with the maximum recorded length being 138 mm in SP accessions. A trend was observed in the correlation between the number of spikes and plant height across all *S. bigelovii* accessions. Two groups of *S. bigelovii* accessions were formed based on spike length (Figure 2a). The first group comprised CCB and G plants, whose spike length varied from 11 to 13 cm on average. Nevertheless, a higher spike number (30 cm on average) was observed for CCB plants, compared to the plant height and to the rest of the accessions.

The root system of a *Salicornia* plant consists of a primary root that originates from the embryo and several lateral or secondary roots that develop after the embryonic stage. *Salicornia* accessions exhibited significant differences in root length (Figure 2b). When irrigated with effluent water, all *Salicornia* accessions developed longer secondary roots compared with their primary roots. The Sp accession plants had the most extensive root systems, both primary and secondary, while the G population had the least developed roots. The BC population displayed the shortest primary roots, yet their secondary roots were the longest. The number of secondary roots (SRN) was notably higher in the SP and SPN accessions compared with other accessions. This root development pattern broadly paralleled seed yield: Sp plants, with the most extensive root systems, also produced the highest seed weight per plant, whereas G, with the least developed roots, produced the lowest. BC was a notable exception, combining the shortest primary roots with the highest field-scale seed yield, indicating that secondary root proliferation rather than primary root length is the trait most closely linked to seed production (see Figure 2e).

Differences in seed weight can occur due to varying environmental conditions across different population locations and/or genetic variations in traits directly affecting seed weight. The seed weight per plant for Sp, SpN, and BC plants exceeded 13.5 g, while the CCB and G accessions had lower weights, ranging from 7 to 8 g per plant (Figure 2c). On average, the seed weight was approximately 10 g per plant. It is well-established that environmental factors significantly impact seed weight. Several factors could lead to differences in seed weight within *S*. *bigelovii* accessions, such as abiotic conditions where the plants grow, which might influence resource availability and reproductive success.

Figure 2d illustrates the seed and biomass yields of *S. bigelovii* accessions. The BC accession recorded the highest yields, with biomass at 195 kg/ha and seed yield at 4344 kg/ha. However, it was noted that BC seeds contained other non-seed materials and debris, indicating lower seed quality and necessitating further processing to clean the seeds. Conversely, the SpN accession had similar seed and biomass yields (193 and 3958 kg/ha, respectively) but with superior seed quality (lower levels of debris and other non-seed material). The remaining accessions exhibited comparable biomass and seed yields. The seed yield data from these accessions grown at the ISEA facility significantly exceeded that of other *S. bigelovii* crops cultivated in other locations reported in the literature [11].

There was significant variation in early germination and flowering times among *Salicornia* accessions. The first to germinate were BC, BCW, and SPN plants. Early flowering was noted in the G accession. However, the time to maturation remained consistent across *Salicornia* accessions. Following a field productivity evaluation of various accessions, we established a baseline for the current productivity of available germplasms and gained a deeper understanding of seed biology. Most accessions from Field crop 2 (2017–2018) exhibited a high germination rate of 99% in the growth chamber. The BC crop, however, had a low germination rate due to seeds not fully maturing. The selected germplasm showed high plant density, confirming a significant emergence rate. Plant density greatly influences competition among plants.

SP and BC were identified as the leading accessions with the highest spike characteristic values. Heatmap clustering of whole-plant traits (Figure 2e) across all accessions indicated a strong correlation between the total number of branches (TBN) and the length of secondary roots (SRL). Seed yield (SW) is directly linked to spike length (SL), with both seed weight and spike length showing a high positive correlation. The length of principal roots (FRL) correlates with the number of secondary roots (SRN), while the total plant length is grouped without any significant correlation to other plant traits. Both the number and length of roots have a direct impact on seed weight. Plant height has low direct correlations with other plant traits but shows a strong positive relationship with fresh biomass production in all *Salicornia* accessions. Among all growth parameters, plant height (FPL) stands out with the highest indirect effect.

### 3.2. Seeds’ Color, Physical Properties, Shape, and Size

**Figure 3:**
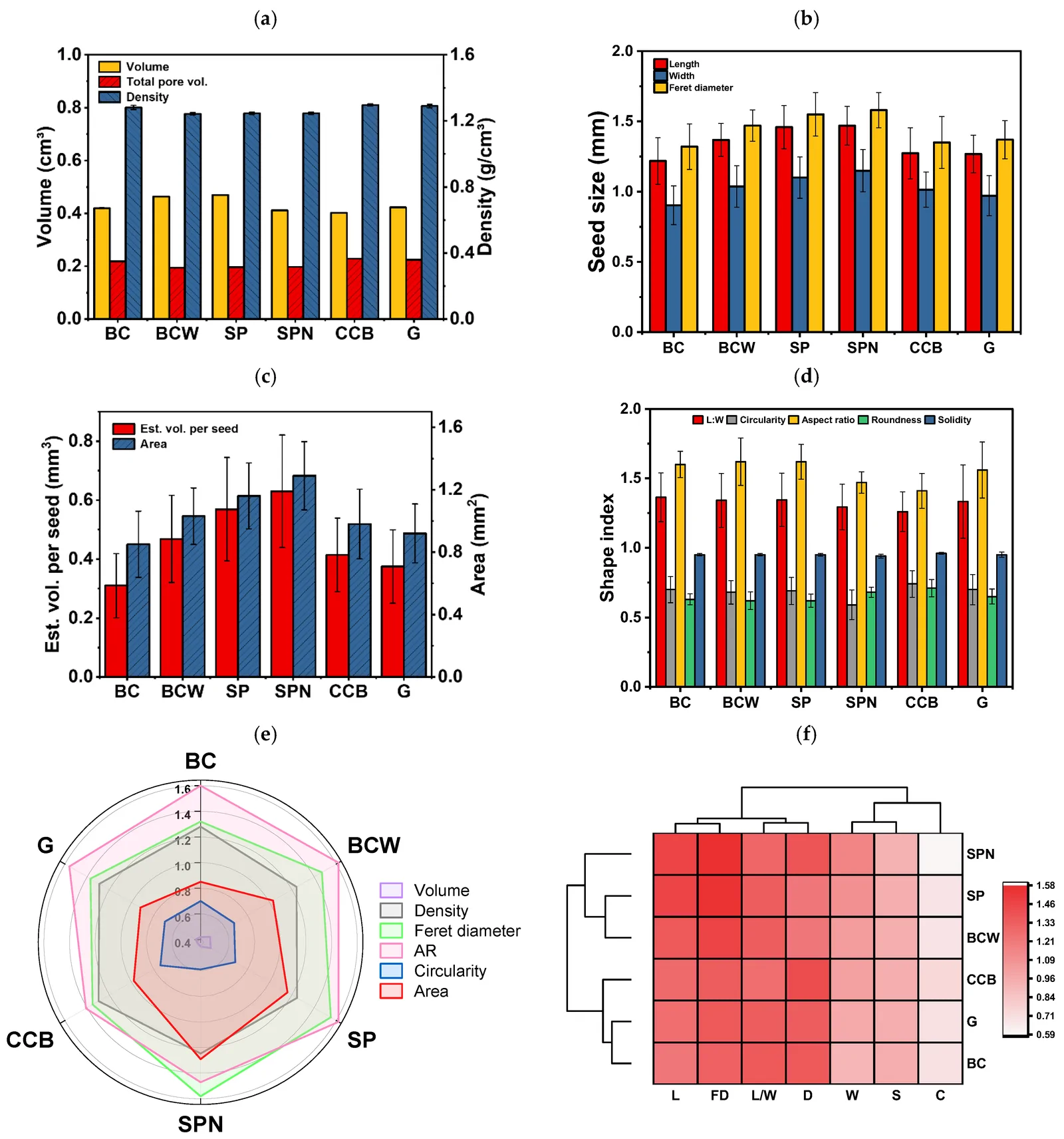
Density, size, and shape descriptors of the *S*. *bigelovii* seed accessions: (**a**) measured volume and density, (**b**) size descriptors, (**c**) area and estimated volume, and (**d**) shape indices; (**e**) radar plot, (**f**) hierarchical clustering of seed shape, size, and physical properties. L: length; FD: Feret diameter, L/W: length to width ratio; D: density; W: width, S: solidity; C: circularity; AR: aspect ratio.

Several studies showed the usefulness of digital image analysis for the phenotyping of plant seeds [33,34]. In this study, quantitative measures of the morphological features (shape and size) of *S*. *bigelovii* seeds were obtained using ImageJ software. The mean values (±SD) of seed size, shape, and physical properties collected for the six *S. bigelovii* accessions are displayed in Figure 3. Statistical comparisons were performed independently for each trait across accessions using one-way ANOVA followed by Tukey’s HSD test (refer to Supplementary Information). All accession-level differences described below for these traits were statistically significant (*p* < 0.05); full mean ± SD values and Tukey grouping letters for every trait and accession are provided in the Supplementary Material. The *S*. *bigelovii* seeds are opaque, ranging between various shades of brown and green, but overall, they are light gold-brown in color as shown in Figure S2.

Physical properties such as density and volume are key to evaluating the quality of the produced seeds. Figure 3a shows the variations in the measured density and volume values between the six accessions of *S*. *bigelovii* seeds. The CCB seeds had the highest density as compared with the other accessions. Conversely, SPN seeds exhibit a comparatively higher volume than the other accessions along with a moderately high density (though lower than CCB), suggesting that they may be of superior quality. While high seed density does not always lead to increased yield, germination rates, seedling growth, or early plant development and maturation, it offers enough benefits to warrant selecting seeds for producing a high-density product. On the other hand, elevated seed density has been observed to lead to markedly slower germination rates. Consequently, under a given set of environmental conditions, excessively high seed density may reduce seedling emergence and establishment due to increased competition for available resources. Studies have demonstrated that seed density can significantly affect seedling emergence, while optimal plant density is dependent on species, seed quality, and environmental conditions [35]. Therefore, the selection criteria for optimal seeds to be used for the breeding program must consider both the seed’s density and germination performance in relationship to the environment in which these seeds will be grown.

Seed descriptors such as length (L) and width (W) ranged between 1.22–1.47 mm and 0.90–1.15 mm, respectively, with the longest being SP accession at 1.5 mm long and the shortest seeds found in BC accession at 1.2 mm long (Figure 3b). Seeds for all accessions are ellipsoid in shape, as depicted by their L:W ratio, with an average ratio of 1.32. The BC accession displayed the largest L:W ratio at 1.4. The pairwise comparison of several size descriptors (Figure S4A–E) displays significant variations, which indicates the morphological dissimilarities observed across accessions, further confirming the variations of *S*. *bigelovii* seeds explained in our earlier work [7].

The estimated volume per seed for all accessions ranged between 0.31 and 0.63 mm^3,^ with the largest volume being SPN with 0.63 mm^3^ and the smallest volume being BC with 0.31 mm^3^ (Figure 3c). The seed surface area ranged between 0.85 and 1.29 mm^2^. Following the same trend, SPN displayed the greatest surface area with 1.3 mm^2^, while BC had the smallest surface area with 0.85 mm^2^. Additionally, seed shape can be defined by several other shape descriptors such as circularity, roundness, and solidity indices as well as AR, which are displayed in Figure 3d. The circularity and AR were evaluated to be between 0.6–0.7 and 1.4–1.6, respectively. SPN seeds were least circular and exhibited the lowest AR compared with the other accessions. The solidity values of all accessions were close to 1, indicating a near-perfect convex shape.

The radar plot (Figure 3e) provides a comparative visualization of the standardized seed morphometric profiles of the six *S. bigelovii* accessions. Overall, SPN seeds had the largest area, whereas BC seeds had the lowest value compared with other seed accessions. CCB seeds were found to have the most rounded seeds with a high circularity index compared with the other accessions, indicating that they are the easiest to clean and process. However, BCW and SP showed more elongation in shape than the other accessions, which might result in increased difficulty in processing.

Hierarchical clustering based on standardized seed morphometric traits using Euclidean distance and the group-average method separated the six *S. bigelovii* accessions into two major phenotypic clusters (Figure 3f). The first cluster comprised SPN, SP, and BCW and the second cluster consisted of CCB, G, and BC, with G and BC forming the closest subgroup. This clustering pattern is consistent with the overall variation observed in the univariate analyses and demonstrates distinct phenotypic differentiation among the evaluated accessions.

The morphometric variables were also grouped into two principal clusters. The first included length, Feret diameter, length/width ratio, and diameter, representing seed size and elongation characteristics, whereas the second comprised width, solidity, and circularity, representing seed shape and compactness. The close association between length and Feret diameter indicates coordinated variation in seed longitudinal dimensions, while the clustering of width, solidity, and circularity reflects shared variation in seed shape. Collectively, these findings indicate that, among the traits evaluated in this study, seed size-related variables contributed strongly to the observed phenotypic variation among the six *S. bigelovii* accessions.

### 3.3. Multivariate Analyses

**Figure 4:**
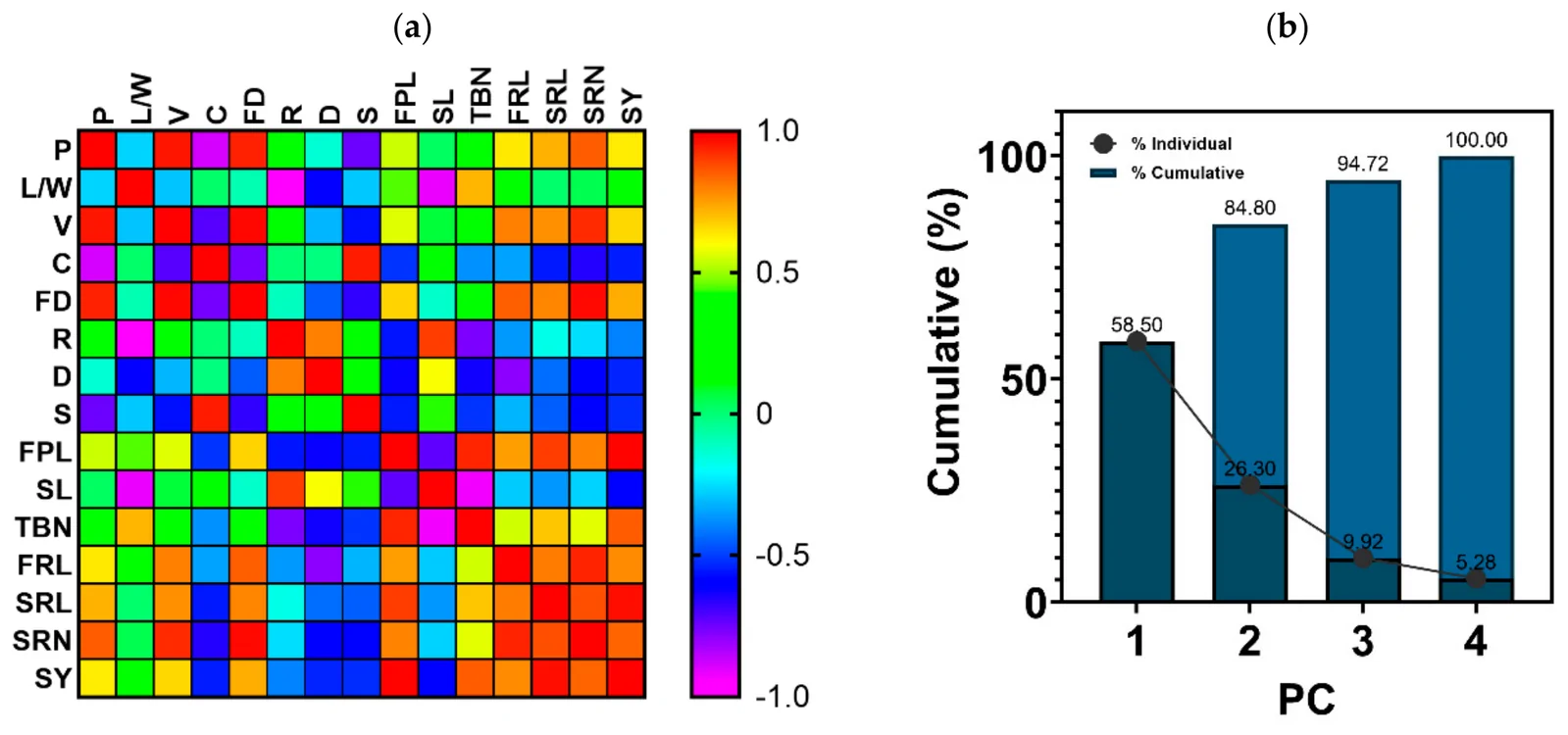

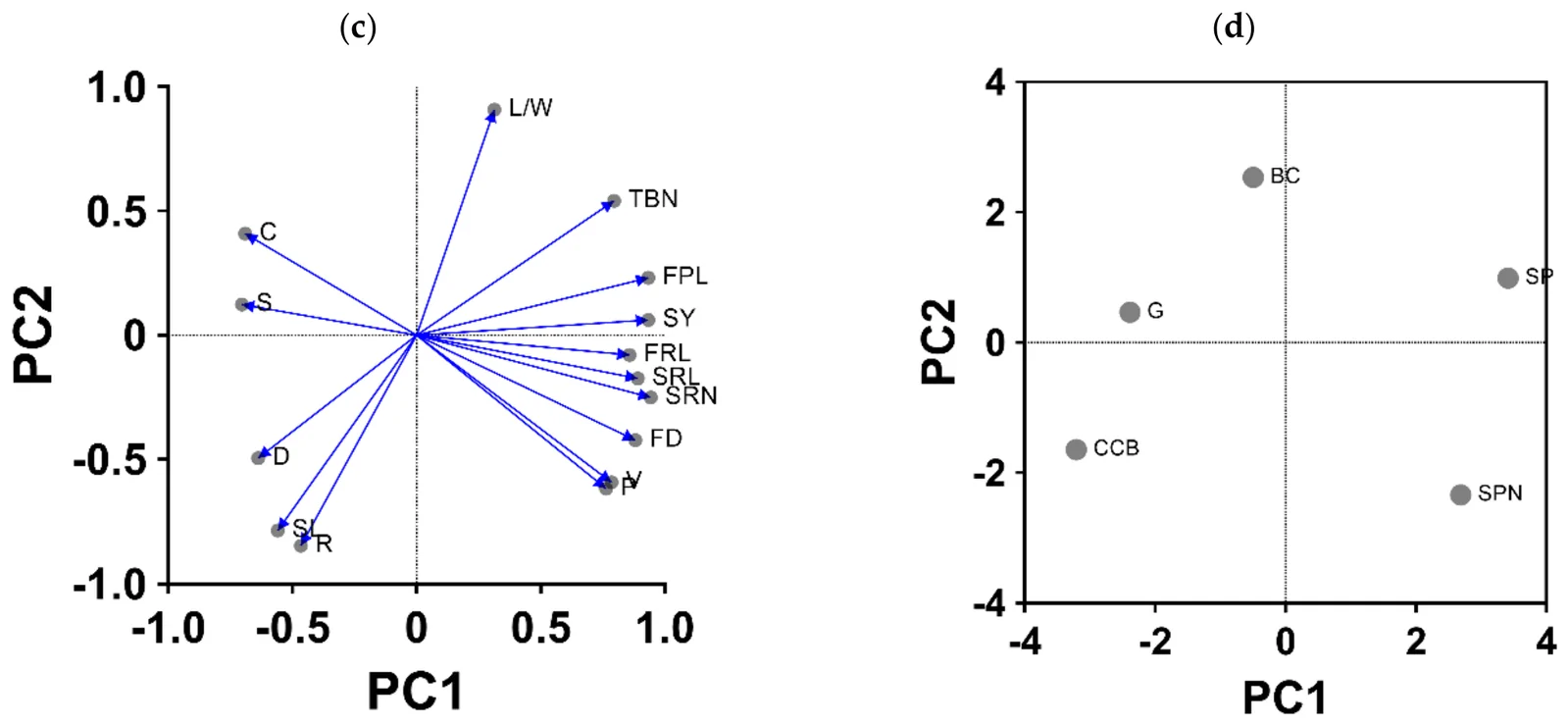
Multivariate analysis of the plant phenotypes and seed parameters of *S*. *bigelovii* accessions: (**a**) Pearson’s correlation coefficients r for 15 morphological traits; red boxes indicate higher levels of correlation, and purple boxes indicate lower levels of correlation; (**b**) variance proportion per PC, (**c**) loading plot, and (**d**) score plot for PC1 and PC2. P: perimeter, V: volume, C: circularity, FD: Feret diameter, R: roundness, D: density, and S: solidity. FPL: full plant length, SL: spike length, TBN: total branch number, FRL: first root length, SRL: secondary root length, SRN: secondary root number, and SY: seed yield.

Multivariate analysis was conducted using Pearson correlation and PCA to elucidate the relationships among the plant phenotypes, seed size, and seed shape descriptors in the different *S*. *bigelovii* seed accessions. Figure 4a presents a heatmap illustrating the Pearson correlation coefficients among the different variables. A strong positive correlation (R ~ 1) was observed between the size descriptors, namely, estimated volume and Feret diameter/perimeter, owing to the dependency on seed length. In addition, the shape indices, namely, circularity and solidity, showed *R* = 0.95. In contrast, a strong negative relationship was observed between L/W and roundness, which is expected because the L/W ratio is a measure of seed elongation.

The correlation structure found in *S. bigelovii* phenotypes demonstrates synchronized patterns of plant vigor, architectural compromises, and allometric relationships that are characteristic of annual species with terminal inflorescences. FPL was strongly positively correlated with TBN (*R* = 0.93), SRL (*R* = 0.90), and SY (*R* = 0.98). This indicates that larger plants are more highly branched and more productive. Such size-dependent integration of vegetative and reproductive traits is widely reported in annuals, where increased shoot growth enhances photosynthetic capacity and root proliferation improves water and nutrient acquisition, ultimately increasing reproductive output [36,37]. In halophytes, biomass accumulation is closely linked to seed yield under saline conditions due to strong source–sink coupling [38]. In this study, FPL served as an indicator of the overall health of the plant.

A strong negative correlation was observed between TBN and SL (*R* = −0.93). In *S. bigelovii*, spikes are terminal structures formed at the end of each branch. Therefore, an increase in branch number increases the number of reproductive meristems competing for limited assimilates. Classical resource allocation and meristem limitation theories predict a trade-off between organ number and organ size under a finite resource supply [39]. Plants producing many branches distribute resources among more terminal spikes, resulting in shorter spikes. This reflects a fundamental architectural constraint in monocarpic species.

Spike length (SL) was strongly positively correlated with seed roundness (*R* = 0.90) and strongly negatively correlated with seed length-to-width ratio (*R* = −0.92). These traits reflect maternal resource allocation and developmental processes affecting seed filling, rather than spike length alone [40,41]. Seed size and shape result from the coordinated growth of the embryo, endosperm/perisperm, and maternal tissues, which is regulated by the maternal control of assimilate supply. In halophytes, the maternal environment, especially salinity, influences carbon balance and carbohydrate availability, thereby impacting seed traits [42,43]. Thus, the correlations of SL indicate variation in maternal provisioning and developmental stability during seed maturation.

Secondary root number (SRN) showed a very strong positive correlation with seed Feret diameter (FD) (*R* = 0.97), indicating that accessions with more proliferated root systems tended to produce larger seeds (FD is a maximum-size seed descriptor). This supports a tight root–reproduction coupling mediated by whole-plant resource acquisition. Functional equilibrium theory predicts that enhanced root proliferation increases the capacity to acquire water and nutrients, which in turn can elevate shoot carbon assimilation and support stronger reproductive provisioning. Halophyte reproduction is significantly influenced by salinity-induced limitations on water dynamics and carbon distribution. Enhancing the capacity for belowground absorption is anticipated to expand the pool of assimilates available for seed development, thereby increasing seed size characteristics such as FD [43,44].

The PCA resulted in a Cartesian plane that explained 84.8% of the total variance in the data, with PC1 and PC2 accounting for 58.5% and 26.3% of the variations, respectively. PC1 and PC2 had eigenvalues of 8.8 and 3.9, respectively (Figure 4b). The principal components revealed patterns of association among plant phenotypic traits and seed descriptors and identified the variables contributing most strongly to the variation among *S. bigelovii* accessions. The loading plot in Figure 4c reveals the influence of 15 different variables on PC1 and PC2. The phenotypes (SRN, SY, TBN, FRL, and SRL) and seed size descriptors (estimated V, P, and FD) exhibited strong positive associations with PC1. In contrast, the L/W ratio demonstrated the strongest positive associations with PC2. This distribution of variables across the principal components provides insights into the key factors driving variability among different *S*. *bigelovii* seed accessions. The PC score plot in Figure 4d reveals three distinct clusters, highlighting similarities and differences among the studied accessions.

The first cluster included *S*. *bigelovii* seed accessions (BC and G), positioned low on PC1 and characterized by smaller seed size (lower estimated V, P, and FD) together with reduced vegetative vigor (lower SRN, SY, TBN, FRL, and SRL); this cluster is less desirable for yield- or size-focused breeding but may retain useful compact-seed or stress-tolerance alleles. The second cluster (Sp) and fourth cluster (SpN) scored highly on PC1, reflecting strong positive associations with root/shoot growth traits and larger seed size descriptors, identifying them as preferred source populations for breeding programs targeting increased seed yield and *Salicornia* biodiesel feedstock biomass. The third cluster (CCB) was distinguished mainly along PC2, driven by its high circularity and density combined with a lower L/W ratio, making it the preferred source for food-grade applications where uniform, compact, easily processed seeds are desirable. This clustering therefore provides a practical roadmap for *S*. *bigelovii* breeders: SP and SPN for yield- and biomass-oriented selection, CCB for seed quality and processing-oriented selection, while BC and G represent a phenotypically distinct group of lower priority for these specific traits. These seed metrics can aid germplasm characterization and may be relevant to seed handling and processing; however, their value as breeding targets would require evidence of heritability and a demonstrated association with agronomically important traits.

The positioning of different *S*. *bigelovii* accessions along the principal components offers a roadmap for valuable agronomic trait improvement in *S*. *bigelovii*, allowing breeders to target specific phenotypes, seed shape, size, or physical property enhancements. This study emphasizes the potential for using various *Salicornia* species interchangeably for food or biofuel production, offering guidance on selecting feedstocks suitable for different applications and environmental settings. Additionally, it highlights the significance of preserving a diverse range of *Salicornia* germplasm, which could lead to more focused research on the genetic and environmental factors that contribute to similarities within each group. These findings lay the groundwork for enhanced breeding programs and cultivation methods aimed at optimizing *Salicornia* and other feedstocks for biofuel production in coastal areas.

### 3.4. Seed Pubescence and Exotestal Ornamentation

**Figure 5:**
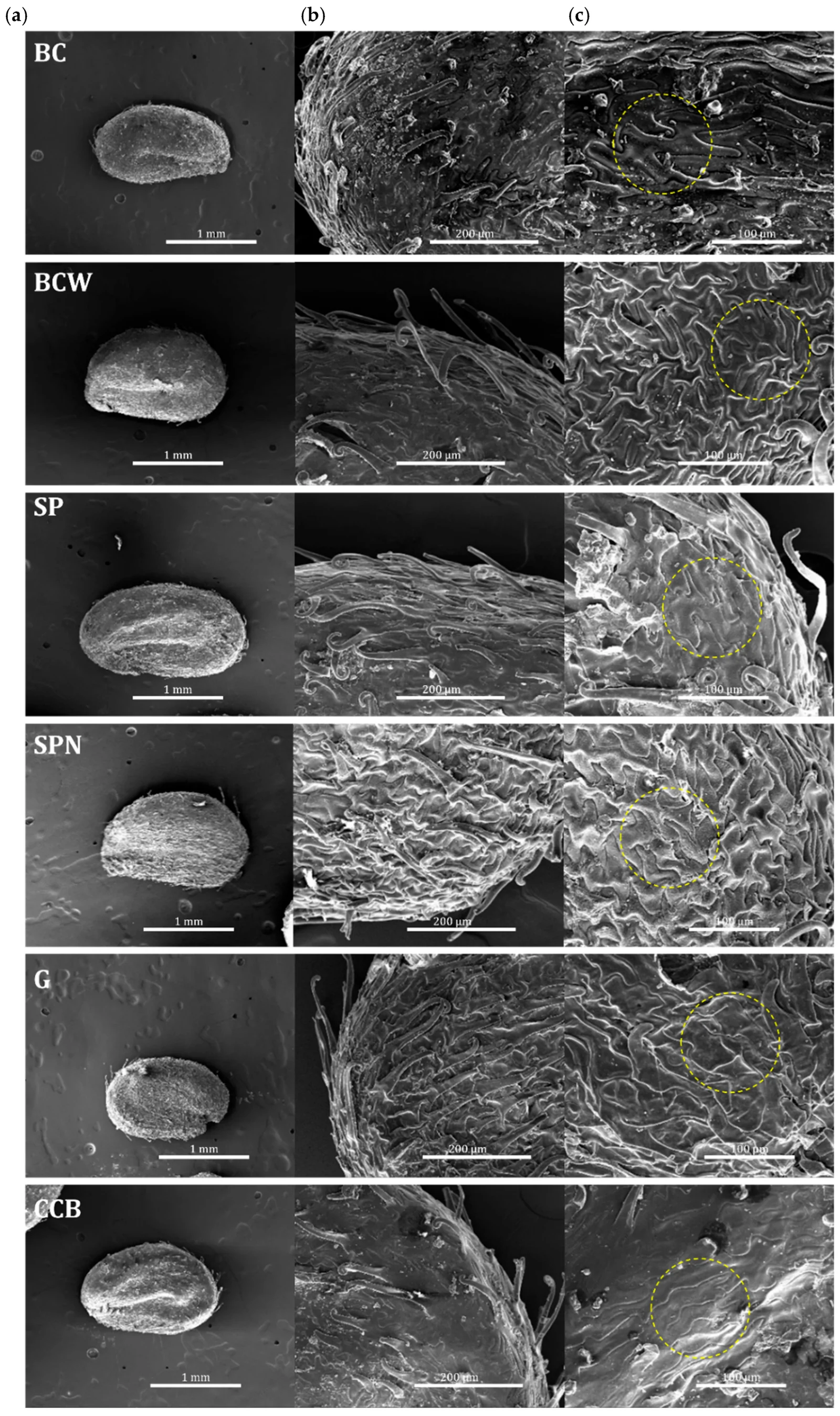
Scanning electron micrographs of *Salicornia bigelovii* accessions showing whole seed, hairs, and seed ornamentation, (**a**) 100×, (**b**) 600×, (**c**) 1000×. The yellow dashed circles show the omega-type contours or jigsaw-puzzle pattern at 1000×.

The micro-morphological features of the seeds were studied using environmental SEM (Figure 5). Seed morphology varied among the accessions, with seed shapes ranging from relatively elongated to more rounded, as shown in Figure 5a. Long and short fine hairs with narrow, acute, and at times a hooked apex were observed in all accessions. Figure 5b shows the uneven elongation of the outer exotesta epidermal cells of the outer integument to form simple hairs in each accession. The ornamentation of the exotesta is not uniform across the seed surface. Seed-coat hairs varied among the accessions in their size and morphology. BC and BCW exhibited relatively short, fine hairs, whereas SP and SPN were characterized by longer hairs with a spiral or coiled morphology. The longer hairs (mean length 172 ± 43 µm) are concentrated around the perimeter of the seed. The seed-coat hairs appeared delicate and were easily ruptured or damaged during sample preparation for ESEM examination (Figure 5b). The view of outer epidermal cells around the hairs along the seed surface showed a crimped omega-type contour or jigsaw-puzzle pattern. At a magnification of 1000×, we observed variations in the microstructure ornamentation of the seed coat surface among the six accessions of *S*. *bigelovii* (Figure 5c). Accessions BC, BCW, SP, and SPN had a similar seed ornamentation type (omega-type contour or jigsaw-puzzle pattern). CCB seeds exhibited very fine, irregular contour lines and less pronounced surface ornamentation than the other accessions. On the other hand, G seeds did not have the omega-type contour, which could be due to the shorter hair covering the seed coat surface of G seeds. This surface sculpturing is biologically significant on two levels: it provides a diagnostic character for distinguishing accessions and taxa within the taxonomically challenging Salicornioideae, and functionally, the interlocking micro-relief of the omega/jigsaw-type pattern can influence water imbibition, mucilage and moisture retention at the seed surface, and adhesion to substrate or dispersal vectors, traits relevant to seed dispersal, sediment retention, and germination timing in the fluctuating saline habitats these halophytes occupy.

#### 3.4.1. Seed Morphology Heteromorphism

Variations in seed characteristics are evident not only between different plant species but also within the same species. This serves as an adaptive mechanism to aid plant survival in environments with high variability, a phenomenon commonly seen in halophytes like *Salicornia* [44,45]. Pairwise comparisons (refer to Supplementary Information) revealed significant differences in seed morphological traits among the *S. bigelovii* accessions. The measured length values were in agreement with previously reported dimensions for *S*. *bigelovii* Torr. (1.6 mm) and *S*. *europaea* L. (1.0–1.7 mm), but they were smaller compared to *S*. *dolichostachya* (1.5–2.3 mm) [25,46]. The measured dimensional values may be useful for the design of seed cleaning equipment (sieving) for the oil extraction process. The measured shape descriptor parameters confirmed that *S*. *bigelovii* seeds are round to ellipsoid in shape, as shown in Figure 3d and SEM micrographs (Figure 5a). The estimated volume was found to be slightly higher than that of *Salicornia patula* (0.11–0.23 mm^3^) [47]. The surface area and volume of seeds are important aspects for the modeling of drying and ventilation seed systems [48]. These seed shape and size characteristics are important for postharvest handling, as seed morphology may influence rolling and sliding behavior during cleaning and can provide useful parameters for designing and optimizing equipment for seed cleaning, transportation, drying, and oil extraction.

#### 3.4.2. Seed Coat Morphology

The heteromorphy of *Salicornia* seeds is well documented in the literature [49,50]. Seed heteromorphism may play a role in changing the seed’s response to selective environmental pressures across a wide geographic range, often providing a more favorable condition for plant survival. Seed coat morphological characteristics are one of the main determinants of seed dormancy, germination, vigor, and longevity, with seed coat structure dissimilarities between plant species and varieties [44,51]. Visual color analysis of the *S*. *bigelovii* seeds shows that they range from light green (G and CCB) to gold-brown (SP and BC). The observed variation in seed color among accessions may reflect differences in the composition and relative abundance of secondary metabolites and other compounds associated with seed pigmentation and development, including phenolic compounds, abscisic acid, and polyamines [24]. The presence of green pigments in mature seeds is not common, although this has been documented for representatives from five other *Chenopodiaceae* genera [12]. It is possible that the *Salicornia* accessions with green-colored seed coats are capable of becoming actively photosynthetic at a very early stage of establishment [25,44].

The seed coat structure data are valuable traits for taxonomic study of the *Salicornia* genus to resolve the generic position of some critical *Salicornideae* [50]. According to the ESEM results, the *S*. *bigelovii* seed coat appears opaque and ornamented with an uneven finger-like projection, or hairs, in some areas (Figure 5b). Our observed seed coat ornamentations stand in close agreement with the *S*. *bigelovii* seed ornamentation observed in [25], where they reported uneven finger-like structures. The hairs present on the seed coat may facilitate seed anchorage, thereby reducing their displacement by tidal water or wave action, which is particularly relevant given that *S. bigelovii* naturally grow along the coastline [24]. We observed that the Southern Site seeds have more surface hairs than those of the Northern Site. This could be due to the fact that the Southern Site along the Texas coastline is exposed to a much harsher and more turbulent environment than the Northern Site.

Despite these qualitative differences in hair density and testa contour type, our SEM analysis did not reveal statistically significant variation across accessions overall, which is consistent with seed coat micromorphology generally being a conserved, canalized trait in Salicornioideae that is more informative for distinguishing genera and species than for differentiating accessions within a single species [52,53,54]. This pattern parallels findings in other plant groups, where seed micromorphological variation observed by SEM does not always track fine-scale phylogenetic, geographic, or environmental differences [52]. In addition, our SEM assessment was primarily qualitative and descriptive rather than based on quantitative measurements subjected to formal statistical testing, unlike the seed morphometric analyses reported above, which limits the ability to detect subtle inter-accession differences. The well-documented heteromorphism of *Salicornia* seeds may further contribute to this pattern, as variability between seeds produced at different positions on the same plant (e.g., from central versus lateral flowers) can be as large as, or larger than, variability between accessions, effectively masking genotype-level differences in a small SEM sample [50].

### 3.5. Geographical Distribution

**Figure 6:**
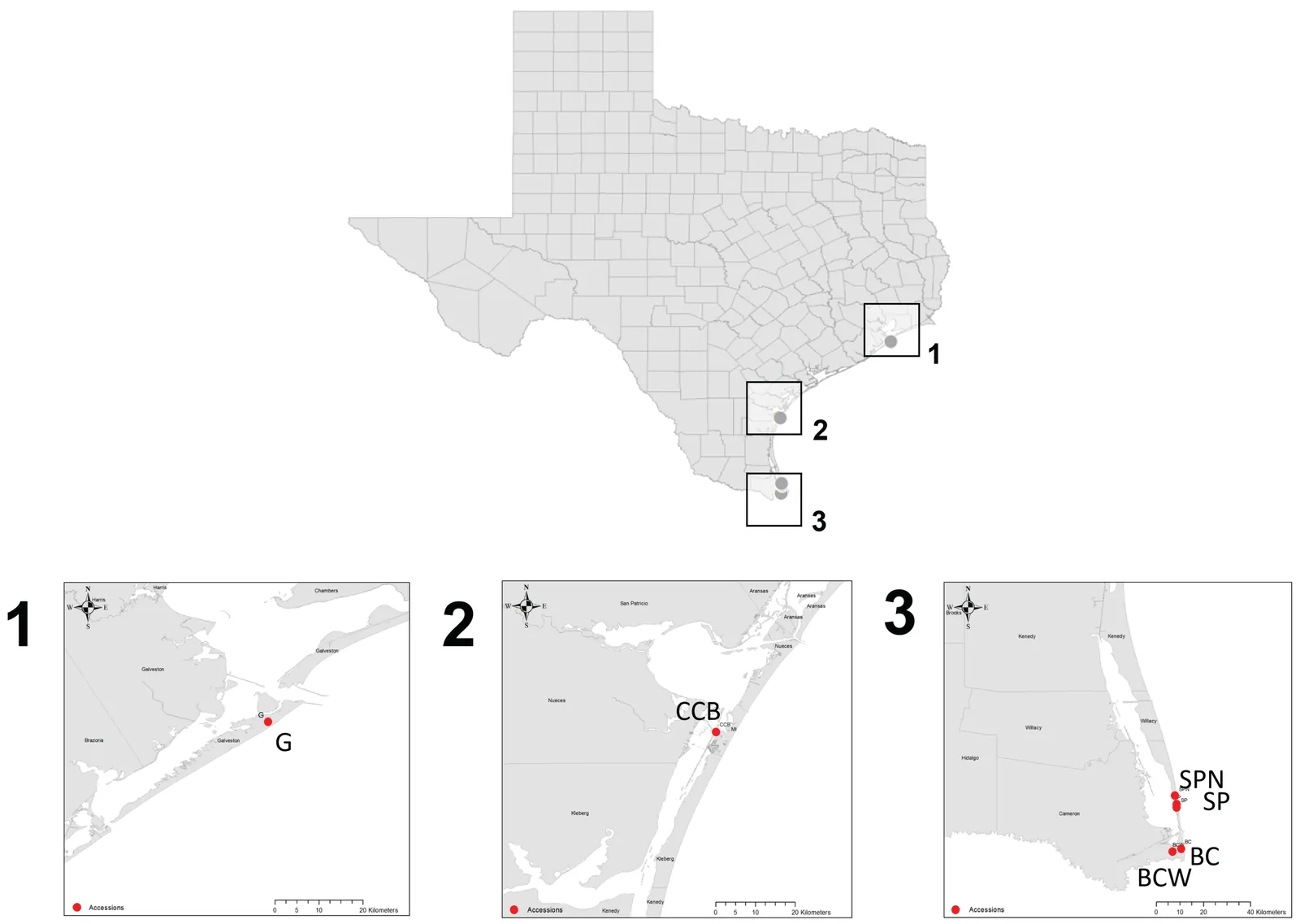
Geospatial distribution of *S*. *bigelovii* accessions along the Texas Gulf Coast. Inserts show zoomed-in locations of (1) CCB, (2) G, and (3) BC, BCW, SP, and SPN accessions.

*S*. *bigelovii* is commonly found along temperate, subtropical, and tropical shorelines [55]. According to the ArcGIS data, the studied *S*. *bigelovii* accessions grow along the Texas coastline on a soil type called Texas Gulf Coast Saline Prairies, which is composed of sediments and/or sand. It has been reported that *S*. *bigelovii* can act as a pioneering species in repopulating saline coastlines after the impact of natural disasters [54]. *S*. *bigelovii* seeds were initially collected at several localities, as shown in Figure 6, representing a wide range of habitats along the Texas coastline, and they were grouped into three geographical sites. Site 1, located furthest to the north, consists of the Galveston accession (G). The soil at this site primarily consists of marine sediments (Soil Surveys by State, NRCS Soils (https://www.usda.gov/)). Site 2 encompassed the Corpus Christi Bridge accession (CCB), whereas the other four accessions—BC, BCS, SP, and SPN—were situated in Site 3, both of which are characterized by sandy soil. All sites are subject to seasonal flooding and hurricanes, but Site 3 accessions are the most affected by these storms because of their location on uninhabited barrier islands. Sampling sites were further categorized into northern sites (Sites 1 and 2) and southern sites (Site 3), which share a coastline with Mexico. It is important to note that the cultivation of *S. bigelovii* on a pilot scale in the UAE was limited to sandy soil.

### 3.6. Sequence Characteristics and Phylogenetic Analysis

**Figure 7:**
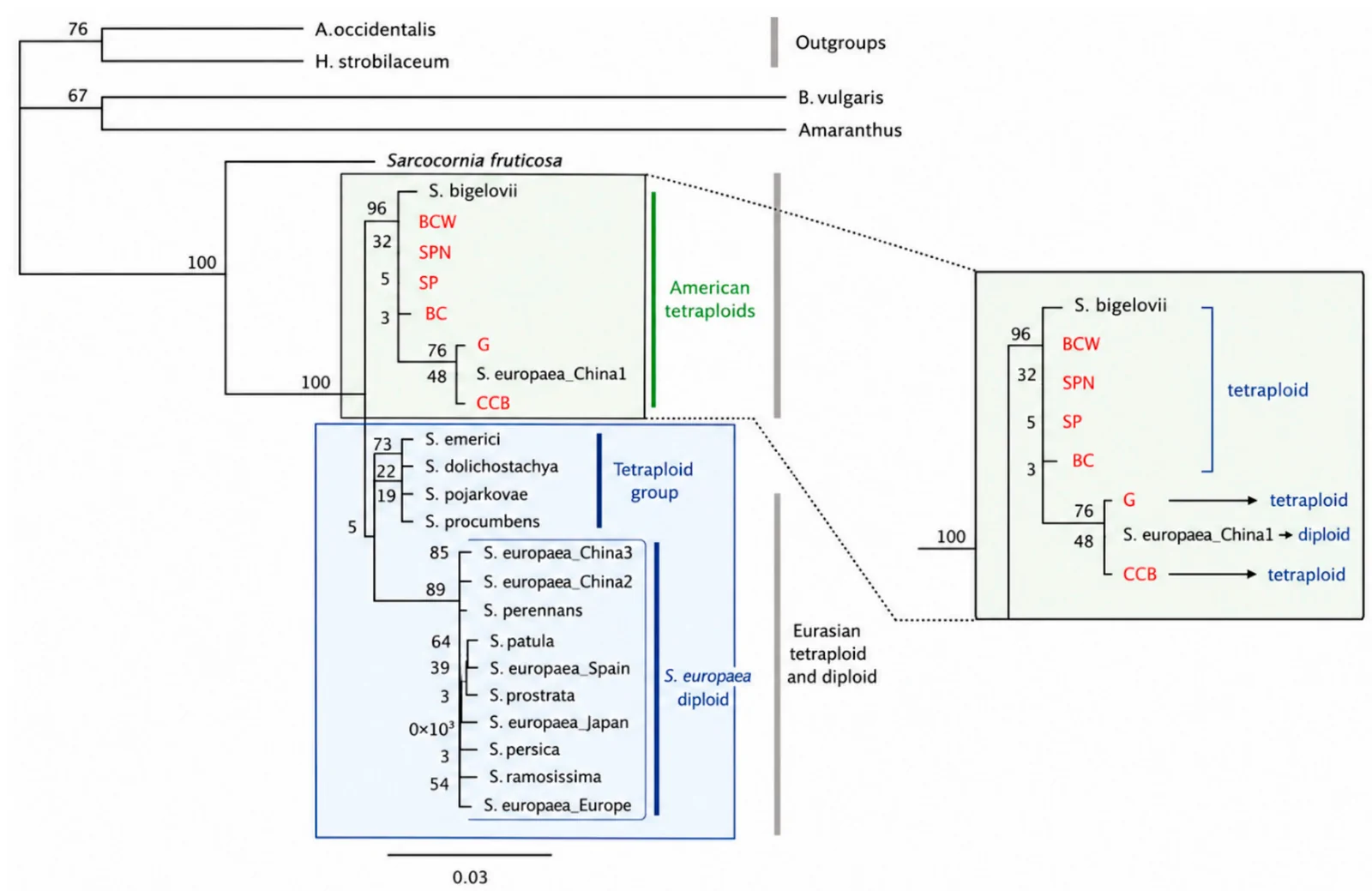
Molecular phylogenetic analyses of *Salicornia* and *Sarcocornia* species. The strict consensus tree inferred from NJ analyses of the ITS regions, with a zoom-out of the new world *Salicornia* species clade extracted from the same NJ tree.

An unrooted ML (refer to Supplementary Information) and NJ phylogenetic tree was constructed with bootstrap analysis (100 replicates) based on the selected ITS sequences mentioned above. Therefore, only branches with bootstrap support ≥70% were considered reliable. The aligned 26 ITS sequences consisted of six accessions of *S*. *bigelovii* seeds described in this work, 17 additional Salicornieae taxa, and four outgroups (*Allenrolfea occidentalis*, *Halocnemum strobilaceum*, *Beta vulgaris*, and *Amaranthus caudatus*). Figure 7 shows the phylogenetic relationships among different genera and species based on the NJ method. All ITS sequence data have been deposited in the NCBI database under the ID number 2521964. The analysis revealed that *Salicornia* was separated from the *Sarcocornia* taxon into two discrete clades, which strongly support the hypothesis that *Salicornia* is a monophyletic group (bootstrap value [BV] =100%). This result is consistent with findings reported in the relevant molecular studies [1,20,22,55]. The *Salicornia* clade diverged into two clades representing American and Eurasian *Salicornia* species (BV = 100%). The Eurasian species were separated into two discrete clades according to their ploidy level (diploid and tetraploid *Salicornia* species) with 89% and 73% BVs, respectively (Figure 7). Variations in the ITS DNA sequences among different *Salicornia* species correspond to their respective geographical origins. The relationships within these groups fall within an unresolved diploid and tetraploid polytomy [19,55]. Moreover, within the diploid clade, two small diploid subclades were revealed: one is a highly supported subclade (BV = 85%) containing *S*. *europaea* from China, and the other is a poorly supported subclade containing *S*. *europaea* and *S*. *ramosissima* (BV = 54%).

The clade comprising mostly tetraploid *S*. *bigelovii* diverged into two subclades (published ITS sequences of *S*. *bigelovii* and ITS sequences of this study). Four of the studied *S*. *bigelovii* accessions, namely, BC, BCW, SP, and SPN, were closely related to the published ITS sequence of *S*. *bigelovii*. Another highly supported subclade (BV = 76%) appears to have evolved later and comprises two *S*. *bigelovii* accessions (G and CCB) and the diploid *S*. *europaea* subspecies geographically located in China. The presence of diploid species within the tetraploid clade in the NJ dendrogram may be attributed to the fact that Chinese *S. europaea* was named after the *S. europaea* species because they appeared to be phenotypically similar. *S. europaea* is one of the most frequent names used in a very broad sense to include most of the species in the genus, which greatly complicates the assignment of published information to taxa of the genus [22]. Another reason for a diploid *S. europeae* species to be placed among tetraploid accessions could be due to hybridization, albeit with a tetraploid mother [22,55]. Further sampling within and between these countries is required to clarify these relationships. Improving the genetic diversity of the collection would require future seed sourcing from these regions in addition to a wide range of other geographic and climatic conditions of the site collection.

The sequences of the ITS region of 6 individuals indicated genetic variation among the studied accessions. The *S*. *bigelovii* ITS region dendrogram (Figure 7, insert figure) suggests two distinct plant clusters (Northern Site, cluster 1: G and CCB, and Southern Site cluster 2: BC, BCW, SP, and SPN). This phylogenetic result is supported by the geographical distribution of the germplasm presented in Figure 6, which forms three distinct clusters (cluster 1: G, cluster 2: CCB, and cluster 3: BC, BCW, SP, and SPN), which is consistent with the literature, but does not prove an association between the ITS genetic variation and the environmental conditions at the original collection sites. Moreover, the phenotypic variation at the micro level had the same pattern as the geographical and genetic variations; because all accessions were subsequently propagated and phenotyped together under common growing conditions rather than at their original sites (Section 2.1 and Section 2.2), this parallel pattern more plausibly reflects genetically fixed differences among accessions—potentially shaped by past local adaptation at their original sites—rather than a direct, real-time effect of the collection-site environment on the phenotypes measured here. The genetic (ITS) divergence itself, however, remains difficult to attribute definitively to geography versus other drivers, as genetic and historical-environmental sources of variation are confounded in the present sampling design (accessions from different sites, assessed with a single molecular marker); their relative contributions cannot be definitively separated with the current dataset. The phenotypic clustering based on seed morphometric traits showed only partial agreement with the ITS-based phylogeny, highlighting that variation in seed morphology cannot be fully explained by a single molecular marker. Future studies employing larger germplasm collections and higher-resolution molecular markers will be essential to better understand genotype–phenotype relationships and support breeding efforts.

Overall, the data suggest that the genetic variations observed in the ITS sequences and differences in plant phenotype could be attributed to genetic drift driven by both geographical distribution and environmental conditions at the sample collection sites. These results are consistent with previous reports of geography-associated genetic and phylogenetic structuring within Salicornieae 23. However, because our sampling design confounds accession identity with collection site, and molecular characterization relied on a single marker, environmental and genetic contributions to this variation cannot be definitively disentangled with the present data. These findings provide a foundation for future research on *S. bigelovii* germplasm, including studies on bioprospecting, greenhouse cultivation, and breeding programs supported by broader genomic resources and additional molecular markers.

This study provides an assessment of phenotypic variation and ITS-based molecular relationships among six *Salicornia bigelovii* accessions. However, the findings should be interpreted in light of several limitations. The molecular analysis was based on a single genetic marker (ITS), which has limited resolution for inferring phylogenetic relationships and genetic diversity among closely related accessions. In addition, the study included a relatively small number of accessions, limiting the representation of the species’ overall genetic diversity. Bootstrap support was estimated using 100 replicates because of computational and software limitations; therefore, only strongly supported branches were interpreted. Future studies incorporating larger and more geographically diverse germplasm collections together with genome-wide molecular markers, ideally under common-garden and multi-environment conditions, will provide a more comprehensive understanding of the genetic diversity, population structure, and breeding potential of *S. bigelovii*.

## 4. Conclusions

*S. bigelovii* is a key halophytic species recognized as one of the most salt-tolerant vascular plants worldwide. In this study, accessions showed significant variation in key traits, such as plant height, spike number, root length, and seed yield. Germplasm accessions, such as SP, SPN, and BC, outperformed CCB and G, indicating strong phenotypic diversity and breeding potential. Specifically, the superior root development and larger seed size of SP and SPN identify them as the most promising parental lines for breeding programs targeting increased seed yield and biodiesel feedstock biomass, while BC’s high field-scale yield supports its use for biomass-oriented selection, provided seed-cleaning steps are optimized. By contrast, CCB’s compact, high-density, high-circularity seeds make it a valuable parental source for improving seed processing and food-grade quality traits, suggesting that crosses between CCB and high-yielding accessions such as SPN could combine yield potential with processing quality. G, despite underperforming in most yield- and size-related traits, showed comparatively early flowering, indicating it may still hold value as a source of early-maturity alleles for short-season breeding strategies rather than being excluded from future breeding efforts. Morphological analysis revealed subtle but distinct differences among the six *S. bigelovii* accessions, with SPN and SP exhibiting superior traits, such as a larger surface area, circularity values nearing 1, and greater seed volume. These traits indicate a higher likelihood of successful germination, efficient dispersal, and favorable flow dynamics. SEM analysis did not reveal significant variation across accessions. Phylogenetic analysis demonstrated genetic divergence of the CCB and G accessions relative to BC, BCW, SP, and SPN. This study represents the first comprehensive morphological and genetic characterization of *S. bigelovii* seed germplasm, offering valuable insights to support the advancement of molecular breeding programs aimed at accelerating the improvement of this species. More specifically, the phenotypic and ITS dataset generated in this study provides an initial foundation for the future development of marker-assisted selection (MAS) approaches in *S. bigelovii*. The phenotypic trait data can support the selection of parental accessions for breeding, while additional genome-wide molecular markers and validation across larger populations will be required to identify robust trait-associated markers that could subsequently accelerate selection in future breeding cycles.

## Figures and Tables

**Table 1:** Characterization of the *S*. *bigelovii* accessions: Geographical coordinates and soil types along the Texas coastline.

ID	KU ID	Name	Location	Soil Category	Soil Map
BC	KUX1	Boca Chica	25°59.873′ N 97°09.607′ W	Sediments, sand	Gulf Coast Saline Prairies
BCW		Boca Chica West	25°59.221′ N 97°11.339′ W	Sediments, sand	Gulf Coast Saline Prairies
CCB	KUX4	Corpus Christi Bridge	37°54.010′ N 97°14.217′ W	Sediments, sand	Gulf Coast Saline Prairies
G	KUX5	Galveston	29°16.867′ N 94°49.550′ W	Mixed, hyperthermic	Gulf Coast Prairie
SP	KUX2	South Padre	26°10.071′ N 97°10.560′ W	Sediments, sand	Gulf Coast Saline Prairies
SPN		South Padre North	26°11.889′ N 97°10.863′ W	Sediments, sand	Gulf Coast Saline Prairies

**Table 2:** Abbreviations of *Salicornia* plant’s traits.

FPL	Full Plant Length
SL	Spike Length
SY	Seed Yield
FRL	First Root Length
SRN	Secondary Root Number
TBN	Total Branches Number
SRL	Secondary Roots Length

## Data Availability

The original contributions presented in this study are included in the article/Supplementary Material. Further inquiries can be directed to the corresponding author.

## Supplementary Materials

The following supporting information can be downloaded at: https://www.mdpi.com/article/10.3390/plants15203210/s1, Figure S1. The general process followed to quantify the morphological characteristics of *S*. *bigelovii* seeds in ImageJ software. A. calibration slide of a black dot (radius = 2500 µm), B. Silhouette image obtained in ImageJ software, C. Algorithm for image analysis and measurement of seed shape profiles. Figure S2 Color variation in *Salicornia* seeds among all Texas accessions. Figure S3 Molecular phylogenetic analyses of *Salicornia* and *Sarcoconia* species. The strict consensus tree inferred from ML analyses of the ITS regions. Figure S4. Pairwise comparison for the seeds’ physical properties, size descriptors, and shape indices. Figure S5. PCA analysis, (a) Eigenvalues, and (b) Biplot. Table S1. Summary of seed size values of different *S*. *bigelovii* seed accessions. Table S2. Summary of seed shape values of different *S*. *bigelovii* seed accessions. Table S3. Summary of seed shape values of different *S*. *bigelovii* seed accessions. Table S4. ITS data matrix including *Salicornia* species and outgroups.
