# Supplementary material for: Phenotypic and Molecular Diversity of Salicornia bigelovii and Its Potential for Salinity-Resilient Industrial Oilseed Production
Source: Plants (Basel). 2026 Oct 19;15(20):3210. doi: 10.3390/plants15203210 (PMC13708847; doi:10.3390/plants15203210)
Supplement: Supplementary file 1 [file plants-15-03210-s001.zip › plants-4564602-supplementary.pdf]

Phenotypic and Molecular Diversity of *Salicornia bigelovii* and Its Potential for Salinity-Resilient Industrial Oilseed Production

Jawaher AlYammahi [a, b, c *], Houda Chelaifa [a, b, e], Bushra G. Mohammed[d], Hector H. Hernandez [b,]

[a] Department of Chemical and Petroleum Engineering, Khalifa University, P.O. Box 127788, Abu Dhabi, **United Arab Emirates (UAE)**

[b] Sustainable Bioenergy Research Consortium, Khalifa University, P.O. Box 127788, Abu Dhabi, **UAE**

[c] Division of Engineering, New York University Abu Dhabi, Abu Dhabi, 129188, **UAE**

[d] Department of Pharmaceutical Chemistry, Shadan Collage of Pharmacy, Hyderabad, **India**

[e] Crop Diversification and Genetics Program, International Center for Biosaline Agriculture (ICBA), Dubai, **UAE**

***Corresponding authors:**

Jawaher AlYammahi (J.alyammahi@nyu.edu)

Supplementary materials

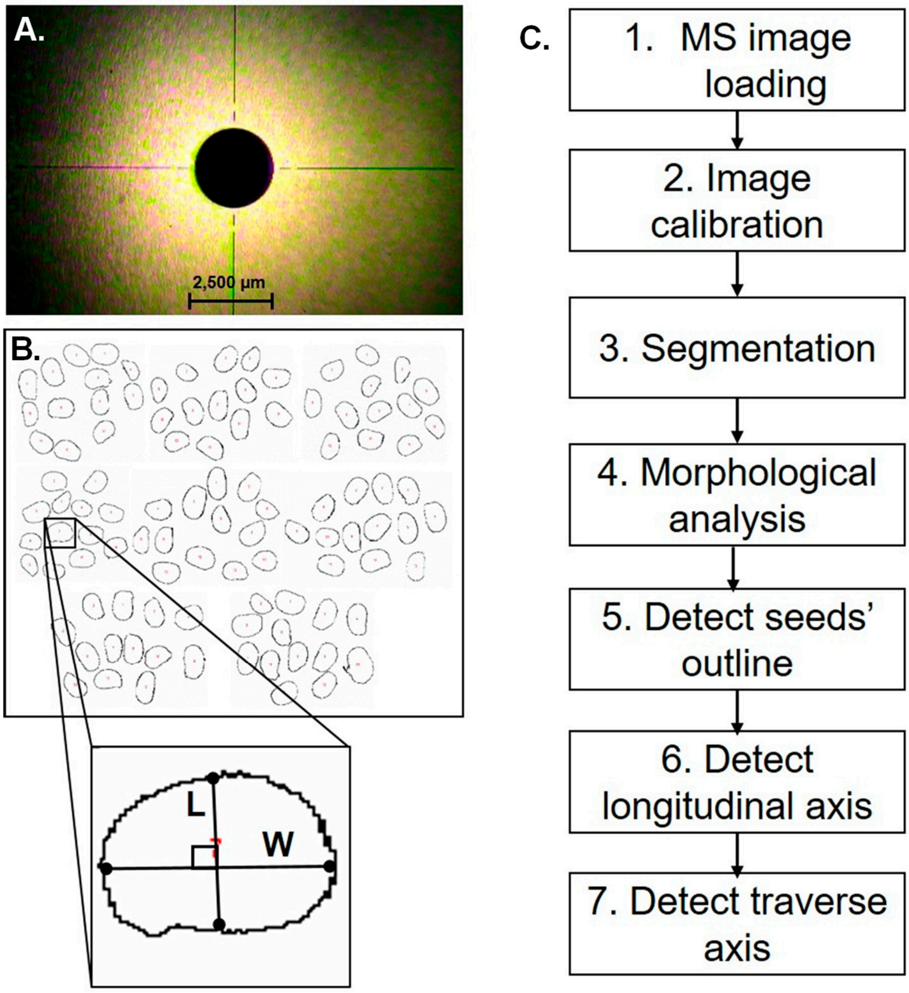

Figure S1. The general process followed to quantify the morphological characteristics of *S. bigelovii* seeds in ImageJ software. A. calibration slide of a black dot (radius=2500 µm), B. Silhouette image obtained in ImageJ software, C. Algorithm for image analysis and measurement of seed shape profiles.

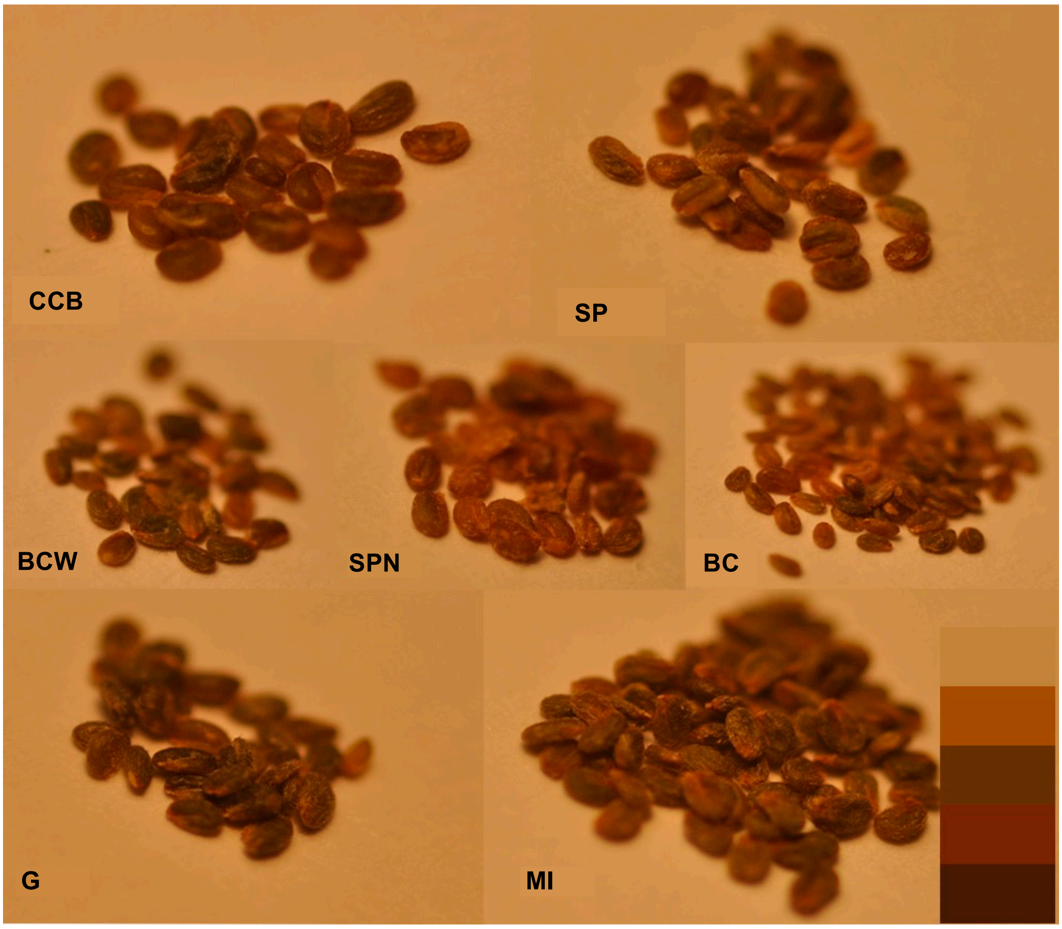

Figure S2 Color variation in *Salicornia* seeds among all Texas accessions.

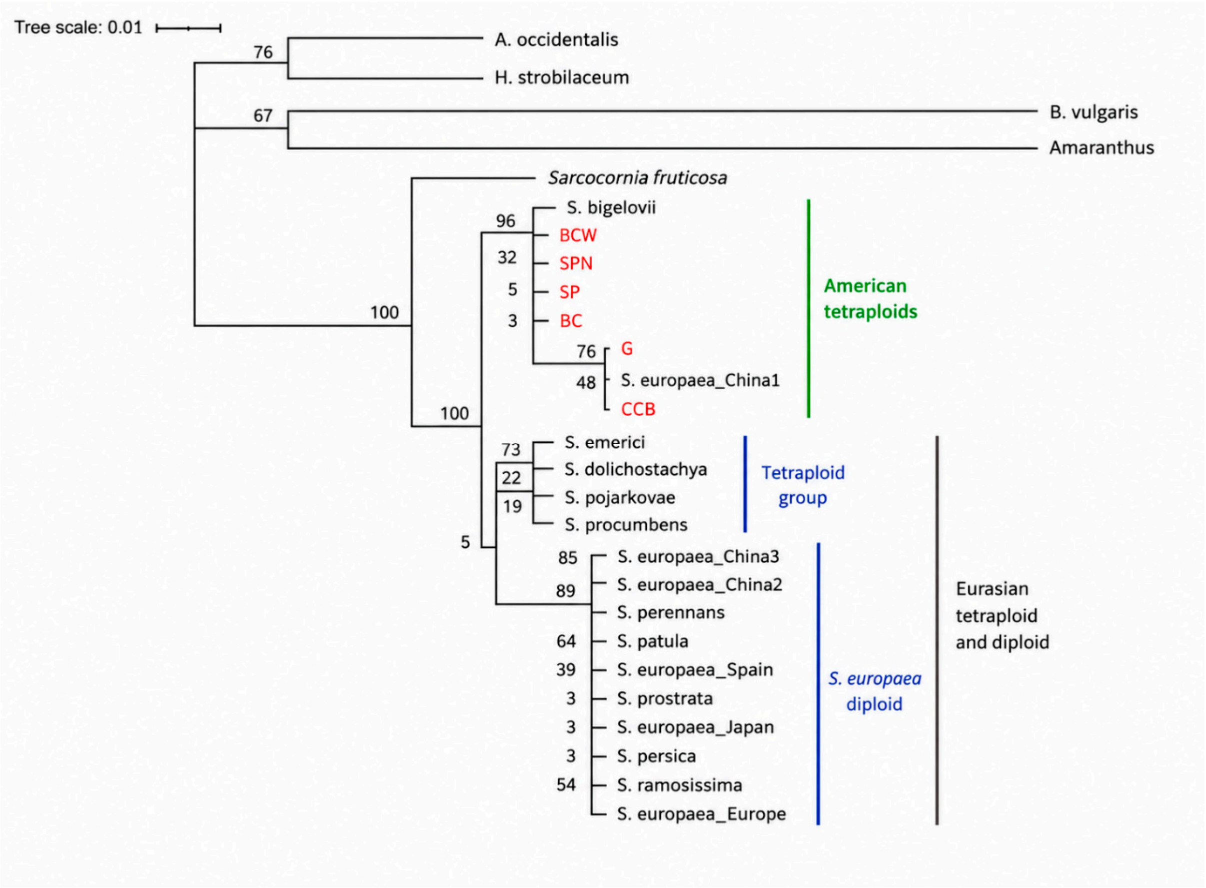

Figure S3 Molecular phylogenetic analyses of *Salicornia* and *Sarcoconia* species. The strict consensus tree inferred from ML analyses of the ITS regions.

Table S1. Summary of seed size values of different *S. bigelovii* seed accessions.

	Seed size				
	**Length (mm)**	**Width (mm)**	**FD* (mm)**	**Perimeter (mm)**	**Area (mm²)**
**BC**	1.218±0.166	0.903±0.138	1.32±0.162	3.88±0.535	0.85±0.212
**BCW**	1.368±0.117	1.037±0.148	1.47±0.111	4.35±0.447	1.03±0.182
**SP**	1.459±0.154	1.100±0.147	1.55±0.154	4.61±0.580	1.16±0.211
**SPN**	1.469±0.137	1.149±0.151	1.58±0.125	5.28±0.701	1.29±0.219
**CCB**	1.273±0.182	1.014±0.125	1.35±0.185	4.08±0.581	0.98±0.224
**G**	1.268±0.134	0.971±0.143	1.37±0.136	4.07±0.528	0.92±0.189

*FD: Feret Diameter.

Table S2. Summary of seed shape values of different *S. bigelovii* seed accessions.

	Seed shape				
	**L/W***	**Circularity**	**Aspect ratio**	**Roundness**	**Solidity**
**BC**	1.364±0.176	0.70±0.094	1.60±0.095	0.63±0.038	0.95±0.009
**BCW**	1.364±0.177	0.68±0.084	1.62±0.171	0.62±0.062	0.95±0.009
**SP**	1.364±0.178	0.69±0.097	1.62±0.126	0.62±0.046	0.95±0.010
**SPN**	1.364±0.179	0.59±0.106	1.47±0.078	0.68±0.035	0.94±0.014
**CCB**	1.364±0.180	0.74±0.095	1.41±0.125	0.71±0.061	0.96±0.007

**G**	1.364±0.181	0.70±0.108	1.56±0.202	0.65±0.053	0.95±0.019

*L/W: Length to width ratio.

Table S3. Summary of seed shape values of different *S. bigelovii* seed accessions

	Physical properties				
	**Mass (g)**	**Volume (cm³)**	**Density (g/cm³)**	**TPV* (cm³/g)**	**Est. vol. (mm³)**
**BC**	0.537	0.41943±0.0040	1.28083±0.012	0.21918±0.0075	0.310±0.108
**BCW**	0.577	0.46413±0.0027	1.2421±0.0073	0.19485±0.0047	0.468±0.148
**SP**	0.585	0.46941±0.0027	1.24606±0.0073	0.19745±0.0047	0.569±0.176
**SPN**	0.513	0.41164±0.0024	1.24629±0.0074	0.1976±0.0047	0.630±0.191
**CCB**	0.521	0.40218±0.0019	1.29645±0.0064	0.22864±0.0038	0.414±0.125
**G**	0.545	0.4225±0.0033	1.29001±0.010	0.22476±0.0061	0.375±0.124

*TPV: Total pore volume; Est. vol.: Estimated volume.

Statistical Analysis (One-way ANOVA)

- **Size Descriptors**

1. Length

Descriptive Statistics

	N Analysis	N Missing	Mean	Standard Deviation	SE of Mean
BC	104	0	1.29993	0.1621	0.01589
BCW	104	0	1.44736	0.11332	0.01111
SP	104	0	1.53394	0.15789	0.01548
SPN	104	0	1.54979	0.13545	0.01328
CCB	104	0	1.31822	0.18592	0.01823
G	104	0	1.34325	0.12684	0.01244

One Way ANOVA

Overall ANOVA

	DF	Sum of Squares	Mean Square	F Value	Prob>F
Model	5	6.35626	1.27125	57.32897	<0.0001
Error	618	13.70395	0.02217		
Total	623	20.0602			

Null Hypothesis: The means of all levels are equal.
Alternative Hypothesis: The means of one or more levels are different.
At the 0.05 level, the population means are significantly different.

2. Width

Descriptive Statistics

	N Analysis	N Missing	Mean	Standard Deviation	SE of Mean
BC	104	0	0.81166	0.11039	0.01082
BCW	104	0	0.8996	0.11053	0.01084
SP	104	0	0.95237	0.09124	0.00895
SPN	104	0	1.05291	0.09844	0.00965
CCB	104	0	0.93194	0.09562	0.00938
G	104	0	0.86683	0.10668	0.01046

One Way ANOVA

Overall ANOVA

	DF	Sum of Squares	Mean Square	F Value	Prob>F
Model	5	3.51857	0.70371	67.0796	<0.0001
Error	618	6.48327	0.01049		
Total	623	10.00183			

3. Feret Diameter

Descriptive Statistics

	N Analysis	N Missing	Mean	Standard Deviation	SE of Mean
BC	104	0	1.32218	0.15728	0.01542
BCW	104	0	1.46785	0.10861	0.01065
SP	104	0	1.54503	0.15403	0.0151
SPN	104	0	1.58152	0.12544	0.0123
CCB	104	0	1.34563	0.18474	0.01811
G	104	0	1.37631	0.13398	0.01314

One Way ANOVA

Overall ANOVA

	DF	Sum of Squares	Mean Square	F Value	Prob>F
Model	5	6.10264	1.22053	57.18112	<0.0001
Error	618	13.19118	0.02134		
Total	623	19.29382			

4. Perimeter

Descriptive Statistics

	N Analysis	N Missing	Mean	Standard Deviation	SE of Mean
BC	104	0	3.91458	0.57803	0.05668
BCW	104	0	4.35708	0.44684	0.04382
SP	104	0	4.55968	0.55294	0.05422
SPN	104	0	5.27531	0.70437	0.06907
CCB	104	0	4.07122	0.57958	0.05683
G	104	0	4.07988	0.52179	0.05117

One Way ANOVA

Overall ANOVA

	DF	Sum of Squares	Mean Square	F Value	Prob>F
Model	5	128.5797	25.71594	79.37677	<0.0001
Error	618	200.21539	0.32397		
Total	623	328.79508			

- **Shape Descriptors**

1. L:W ratio

Descriptive Statistics

	N Analysis	N Missing	Mean	Standard Deviation	SE of Mean
BC	104	0	1.60755	0.10234	0.01003
BCW	104	0	1.62494	0.17042	0.01671
SP	104	0	1.61485	0.12857	0.01261
SPN	104	0	1.4748	0.07813	0.00766
CCB	104	0	1.41329	0.12127	0.01189
G	104	0	1.56483	0.20339	0.01994

One Way ANOVA

Overall ANOVA

	DF	Sum of Squares	Mean Square	F Value	Prob>F
Model	5	3.9207	0.78414	39.79623	<0.0001
Error	618	12.17698	0.0197		
Total	623	16.09768			

2. Circularity

Descriptive Statistics

	N Analysis	N Missing	Mean	Standard Deviation	SE of Mean
BC	104	0	0.69298	0.11191	0.01097
BCW	104	0	0.68178	0.08445	0.00828
SP	104	0	0.70288	0.09148	0.00897
SPN	104	0	0.59414	0.10694	0.01049
CCB	104	0	0.74178	0.09422	0.00924
G	104	0	0.70282	0.10826	0.01062

One Way ANOVA

Overall ANOVA

	DF	Sum of Squares	Mean Square	F Value	Prob>F
Model	5	1.26705	0.25341	25.31612	<0.0001
Error	618	6.18609	0.01001		
Total	623	7.45314			

3. Aspect ratio

Descriptive Statistics

	N Analysis	N Missing	Mean	Standard Deviation	SE of Mean
BC	104	0	1.60756	0.10234	0.01004
BCW	104	0	1.62493	0.17044	0.01671
SP	104	0	1.61486	0.12861	0.01261
SPN	104	0	1.47481	0.07817	0.00766
CCB	104	0	1.41333	0.12124	0.01189
G	104	0	1.56476	0.20338	0.01994

One Way ANOVA

Overall ANOVA

	DF	Sum of Squares	Mean Square	F Value	Prob>F
Model	5	3.91927	0.78385	39.77658	<0.0001
Error	618	12.17858	0.01971		
Total	623	16.09785			

4. Roundness

Descriptive Statistics

	N Analysis	N Missing	Mean	Standard Deviation	SE of Mean
BC	104	0	0.62454	0.03948	0.00387
BCW	104	0	0.62171	0.06106	0.00599
SP	104	0	0.62291	0.04676	0.00459
SPN	104	0	0.67985	0.0351	0.00344
CCB	104	0	0.71254	0.05909	0.00579
G	104	0	0.64537	0.0527	0.00517

One Way ANOVA

Overall ANOVA

	DF	Sum of Squares	Mean Square	F Value	Prob>F
Model	5	0.72775	0.14555	58.31897	<0.0001
Error	618	1.54237	0.0025		
Total	623	2.27012			

5. Solidity

Descriptive Statistics

	N Analysis	N Missing	Mean	Standard Deviation	SE of Mean
BC	104	0	0.95018	0.0111	0.00109
BCW	104	0	0.95075	0.00915	8.97027E-4
SP	104	0	0.95027	0.01	9.80127E-4
SPN	104	0	0.93853	0.01363	0.00134
CCB	104	0	0.95651	0.00642	6.29585E-4
G	104	0	0.94648	0.01956	0.00192

One Way ANOVA

Overall ANOVA

	DF	Sum of Squares	Mean Square	F Value	Prob>F
Model	5	0.01853	0.00371	24.27029	<0.0001
Error	618	0.09437	1.52708E-4		
Total	623	0.1129			

Physical properties

1. Density

Descriptive Statistics

	N Analysis	N Missing	Mean	Standard Deviation	SE of Mean
BC	10	0	1.28083	0.01232	0.0039
BCW	10	0	1.2421	0.00738	0.00233
SP	10	0	1.24606	0.00738	0.00234
SPN	10	0	1.24629	0.00741	0.00234
CCB	10	0	1.29645	0.00641	0.00203
G	10	0	1.29001	0.0102	0.00323

One Way ANOVA

Overall ANOVA

	DF	Sum of Squares	Mean Square	F Value	Prob>F
Model	5	0.03075	0.00615	80.06028	<0.0001
Error	54	0.00415	7.68276E-5		
Total	59	0.0349			

2. Volume

Descriptive Statistics

	N Analysis	N Missing	Mean	Standard Deviation	SE of Mean
BC	10	0	0.41943	0.00405	0.00128
BCW	10	0	0.46413	0.00276	8.72296E-4
SP	10	0	0.46941	0.00278	8.78692E-4
SPN	10	0	0.41164	0.00245	7.74051E-4
CCB	10	0	0.40218	0.00197	6.2179E-4
G	10	0	0.4225	0.00336	0.00106

One Way ANOVA

Overall ANOVA

	DF	Sum of Squares	Mean Square	F Value	Prob>F
Model	5	0.03983	0.00797	903.90667	<0.0001
Error	54	4.75851E-4	8.81206E-6		
Total	59	0.0403			

3. Total pore volume

Descriptive Statistics

	N Analysis	N Missing	Mean	Standard Deviation	SE of Mean
BC	10	0	0.21918	0.00753	0.00238
BCW	10	0	0.19485	0.00477	0.00151
SP	10	0	0.19745	0.00476	0.00151
SPN	10	0	0.1976	0.00478	0.00151
CCB	10	0	0.22864	0.00381	0.00121
G	10	0	0.22476	0.00617	0.00195

One Way ANOVA

Overall ANOVA

	DF	Sum of Squares	Mean Square	F Value	Prob>F
Model	5	0.01189	0.00238	80.36611	<0.0001
Error	54	0.0016	2.9598E-5		
Total	59	0.01349			

4. Estimated volume

Descriptive Statistics

	N Analysis	N Missing	Mean	Standard Deviation	SE of Mean
BC	104	0	0.27996	0.10824	0.01061
BCW	104	0	0.37967	0.11323	0.0111
SP	104	0	0.45425	0.12874	0.01262
SPN	104	0	0.56895	0.15935	0.01563
CCB	104	0	0.37376	0.12979	0.01273
G	104	0	0.32686	0.1073	0.01052

One Way ANOVA

Overall ANOVA

	DF	Sum of Squares	Mean Square	F Value	Prob>F
Model	5	5.43966	1.08793	68.81106	<0.0001
Error	618	9.77084	0.01581		
Total	623	15.2105			

Pair-wise comparison

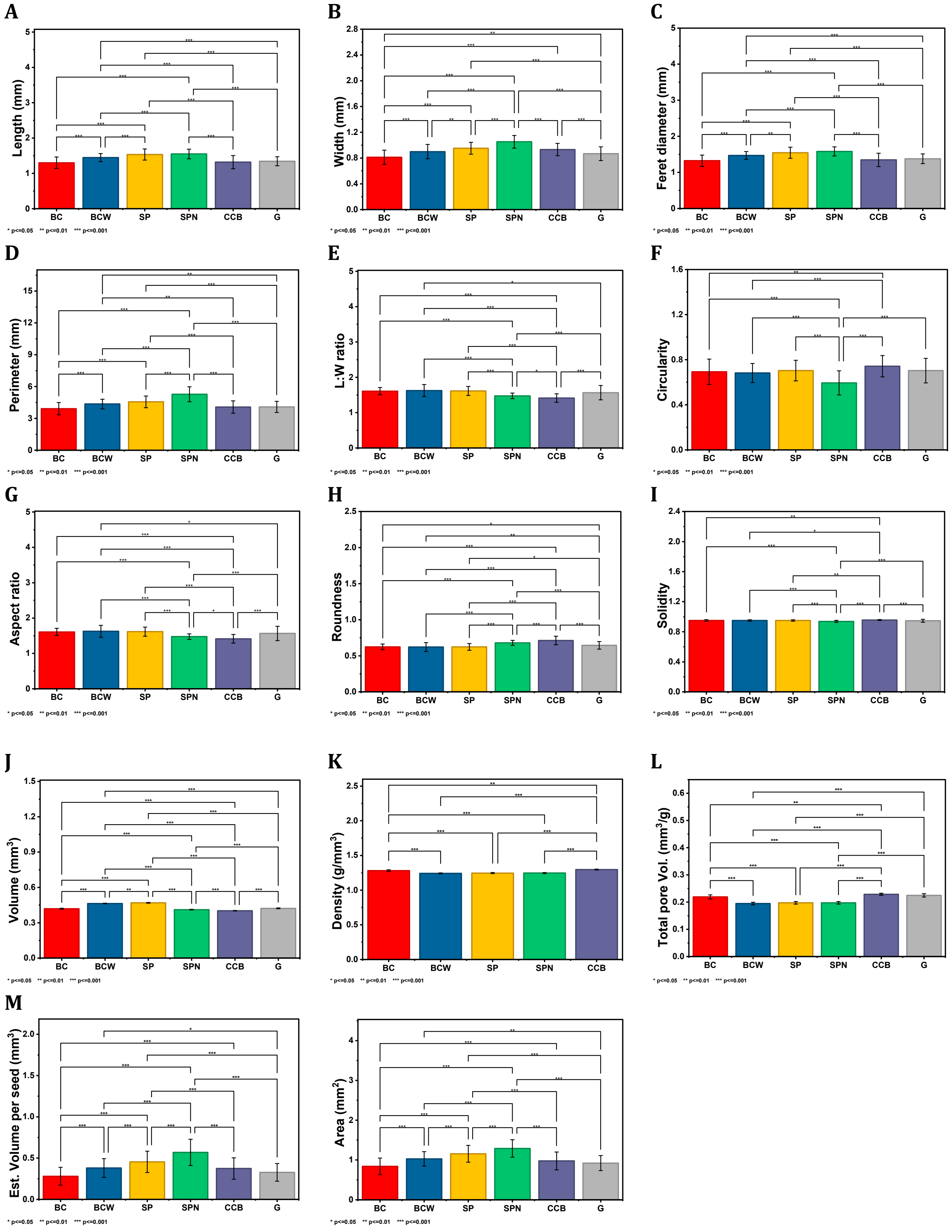

Figure S4. Pairwise comparison for the seeds' physical properties, size descriptors, and shape indices.

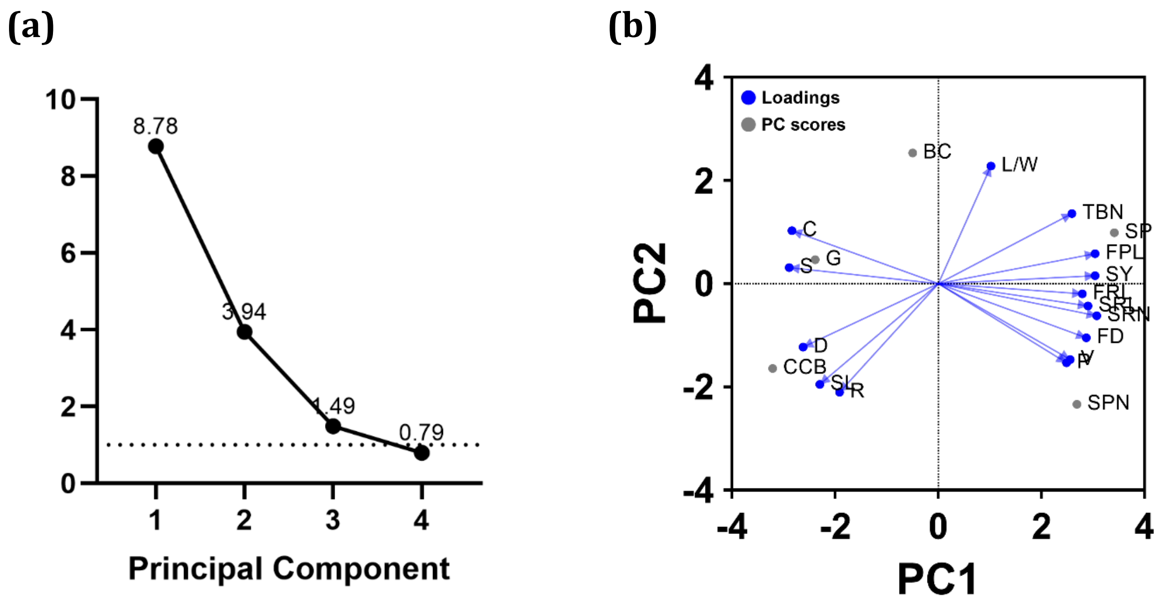

Figure S5. PCA analysis, (a)Eigenvalues, and (b)Biplot.

Table S4. ITS data matrix including *Salicornia* species and outgroups.

Taxon	GenBank accession no.	Phylogenetic tree abbreviation
Allenrolfea occidentalis	AY181875	*A. occidentalis*
Halocnemum strobilaceum	AJ577568	*H. strobilaceum*
Beta vulgaris	AY858597	*B. vulgaris*
Amaranthus caudatus	KX090193	*Amaranthus*
Salicornia europaea	AB537514	*S. europaea* _Japan
Salicornia europaea	MF063453	*S. europaea*_China1
Salicornia europaea	AY489247	*S. europaea*_Europe
Salicornia europaea	MF063455	*S. europaea*_China2
Salicornia europaea	AY181880	*S. europaea*_Spain
Salicornia europaea	MF063456	*S. europaea*_China3
Salicornia bigelovii Torr.	EU682686	*S. bigelovii*
Sarcocornia fruticosa	KU748558	*S. fruticosa*
Salicornia emerici	EF455624	*S. emerici*
Salicornia persica	EF453460	*S. persica*
Salicornia perennans	AY489249	*S. perennans*
Salicornia patula	EF455625	*S. patula*
Salicornia pojarkovae	DQ340158	*S. pojarkovae*
Salicornia ramosissima	AY996251	*S. ramosissima*
Salicornia procumbens	AY489250	*S. procumbens*
Salicornia prostrata	AY489251	*S. prostrata*
Salicornia dolichostachya	AY996234	*S. dolichostachya*

Alignment Results

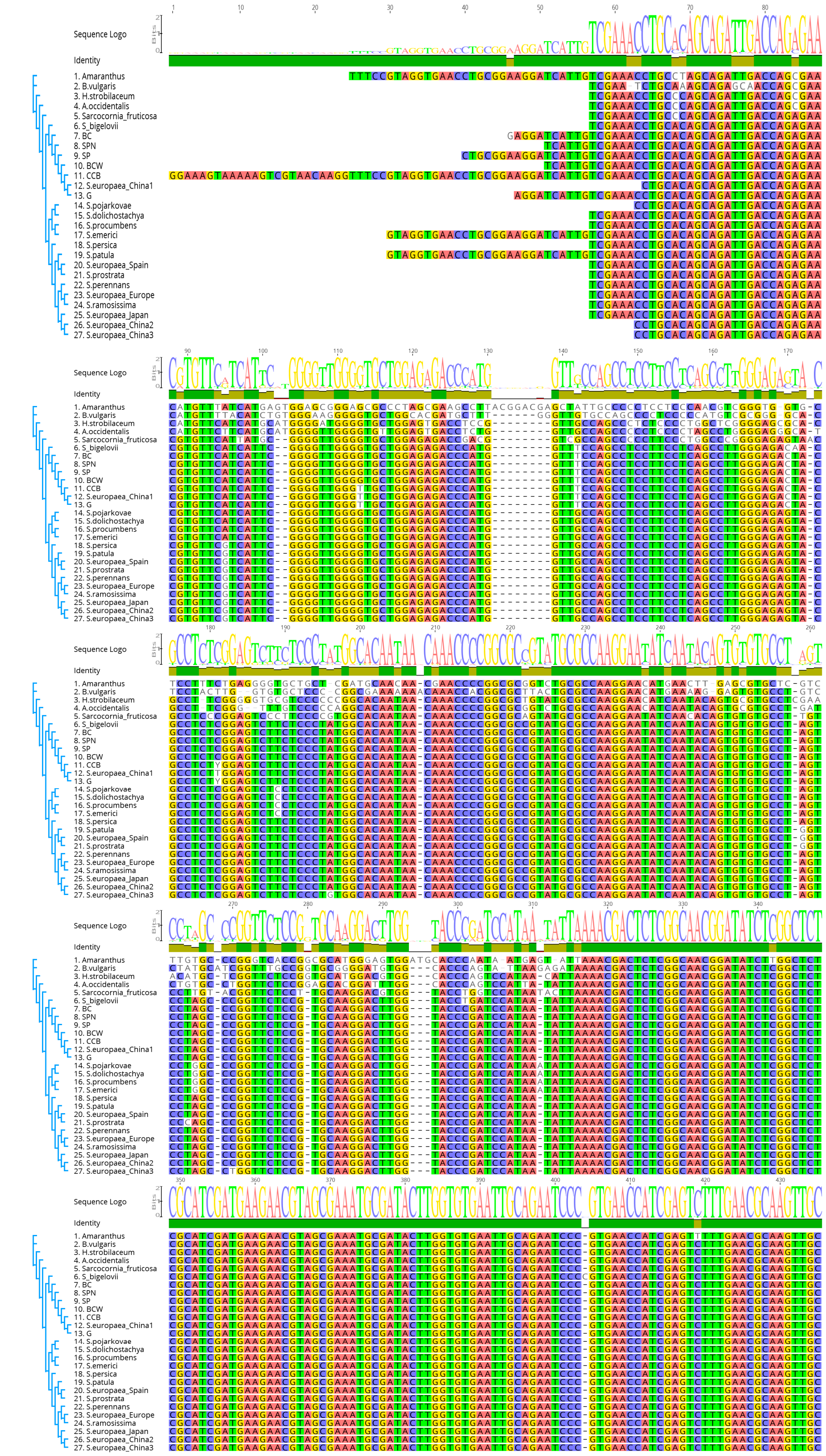

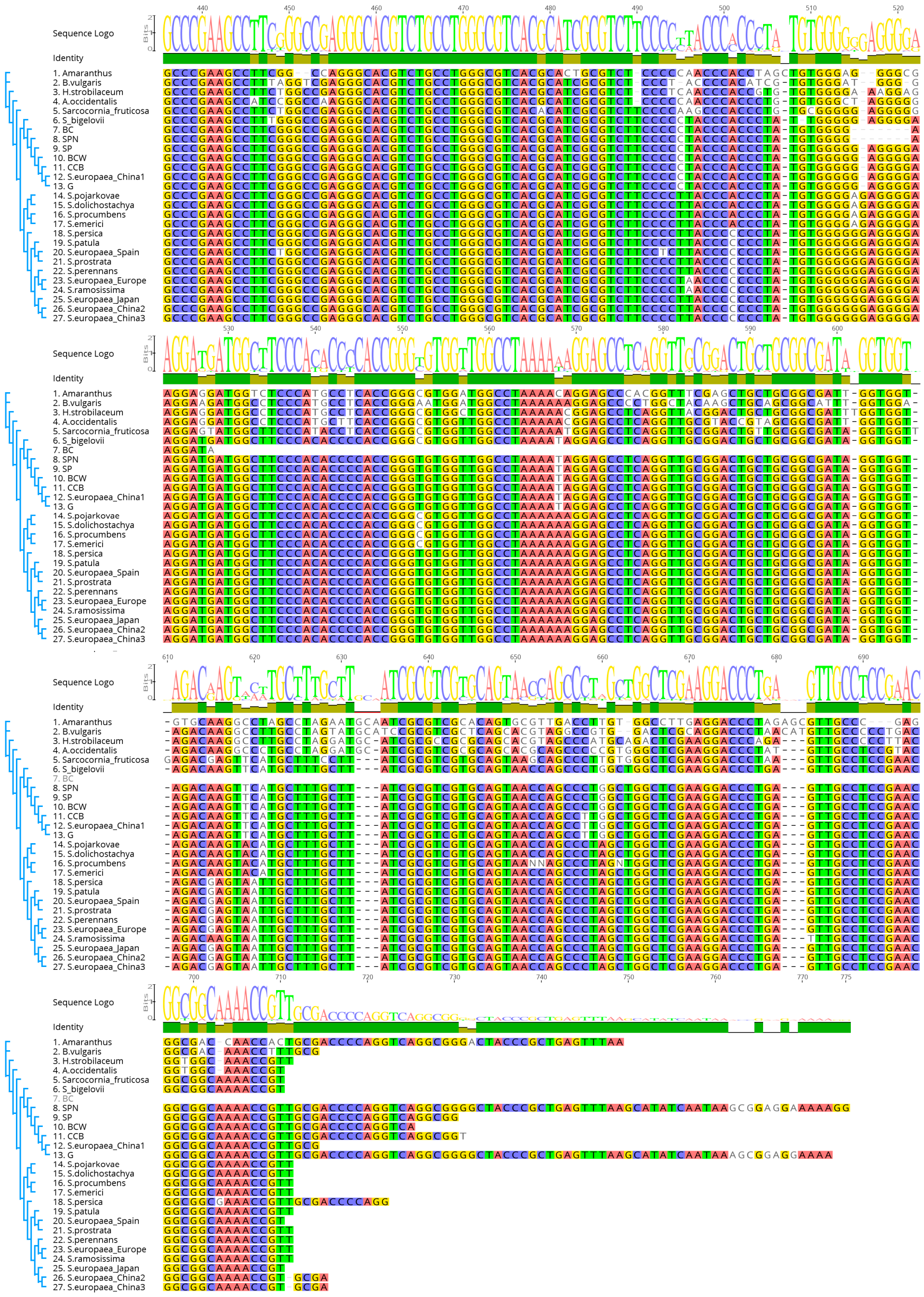

Sequence Logo
Identity
1. Amaranthus
2. B.vulgaris
3. H.strobilaceum
4. A.occidentalis
5. Sarcocornia_fruticosa
6. S_bigelovii
7. BC
8. SPN
9. SP
10. BCW
11. CCB
12. S.europaea_China1
13. G
14. S.pojarkovae
15. S.dolichostachya
16. S.procumbens
17. S.emerici
18. S.persica
19. S.patula
20. S.europaea_Spain
21. S.prostrata
22. S.perennans
23. S.europaea_Europe
24. S.ramosissima
25. S.europaea_Japan
26. S.europaea_China2
27. S.europaea_China3
